# Immune environment and antigen specificity of the T cell receptor repertoire of malignant ascites in ovarian cancer

**DOI:** 10.1371/journal.pone.0279590

**Published:** 2023-01-06

**Authors:** Kyoko Yoshida-Court, Tatiana V. Karpinets, Aparna Mitra, Travis N. Solley, Stephanie Dorta-Estremera, Travis T. Sims, Andrea Y. Delgado Medrano, Molly B. El Alam, Mustapha Ahmed-Kaddar, Erica J. Lynn, K. Jagannadha Sastry, Jianhua Zhang, Andrew Futreal, Alpa Nick, Karen Lu, Lauren E. Colbert, Ann H. Klopp

**Affiliations:** 1 Department of Radiation Oncology, The University of Texas MD Anderson Cancer Center, Houston, Texas, United States of America; 2 Department of Genomic Medicine, The University of Texas MD Anderson Cancer Center, Houston, Texas, United States of America; 3 Comprehensive Cancer Center, Cancer Biology, Department of Microbiology and Zoology, University of Puerto Rico, Medical Sciences Campus, San Juan, Puerto Rico; 4 Department of Gynecologic Oncology and Reproductive Medicine, The University of Texas MD Anderson Cancer Center, Houston, Texas, United States of America; 5 Department of Thoracic/Head and Neck Medical Oncology, The University of Texas MD Anderson Cancer Center, Houston, Texas, United States of America; 6 Saint Thomas Health/Ascension, Nashville, TN, United States of America; 7 Tennessee Oncology, Nashville, Tennessee, United States of America; University of Virginia, UNITED STATES

## Abstract

We evaluated the association of disease outcome with T cell immune-related characteristics and T cell receptor (TCR) repertoire in malignant ascites from patients with high-grade epithelial ovarian cancer. Ascitic fluid samples were collected from 47 high-grade epithelial ovarian cancer patients and analyzed using flow cytometry and TCR sequencing to characterize the complementarity determining region 3 TCR β-chain. TCR functions were analyzed using the McPAS-TCR and VDJ databases. TCR clustering was implemented using Grouping of Lymphocyte Interactions by Paratope Hotspots software. Patients with poor prognosis had ascites characterized by an increased ratio of CD8+ T cells to regulatory T cells, which correlated with an increased productive frequency of the top 100 clones and decreased productive entropy. TCRs enriched in patients with an excellent or good prognosis were more likely to recognize cancer antigens and contained more TCR reads predicted to recognize epithelial ovarian cancer antigens. In addition, a TCR motif that is predicted to bind the *TP53* neoantigen was identified, and this motif was enriched in patients with an excellent or good prognosis. Ascitic fluid in high-grade epithelial ovarian cancer patients with an excellent or good prognosis is enriched with TCRs that may recognize ovarian cancer-specific neoantigens, including mutated *TP53* and *TEAD1*. These results suggest that an effective antigen-specific immune response in ascites is vital for a good outcome in high-grade epithelial ovarian cancer.

## Introduction

Ovarian cancer is the fifth most common cause of cancer death among women in the United States as of 2017 [1]. Most ovarian cancers are high-grade serous ovarian carcinoma (HGSOC) [2], and most patients with HGSOC present with malignant ascites upon initial diagnosis [2, 3]. Treatment is a combination of surgical resection with platinum-based chemotherapy [4]. Traditionally, disease recurring 6 months or more after the last platinum-based treatment is considered platinum-sensitive, and disease recurring less than 6 months after a platinum-based treatment is considered platinum-resistant [5].

The presence of tumor-infiltrating lymphocytes within primary ovarian tumors is associated with high survival rates [6, 7]. However, similar analyses of lymphocytes in the ascites have been more limited [8, 9], even though the ascitic fluid is easy to access. If the immune environment of ascites contributes to ovarian cancer progression, the characteristics of ascites might have important prognostic value, independent of primar tumor clinicopathologic characteristics. Sequencing of hypervariable complementarity determining region 3 (CDR3) regions of the T cell receptor (TCR) repertoire in ascites might provide insights into the immune repertoire of T cells associated with favorable prognosis and identify putative epitopes that could be used to develop ovarian cancer vaccines.

To determine whether the immune environment of ascites contributes to ovarian cancer outcomes, we analyzed the association of immune cell functions and global TCR repertoire characteristics in ascitic fluid T cells with overall survival (OS) and response to therapy in 47 patients with high-grade epithelial ovarian cancer. To provide deeper insight into specific TCR peptides associated with good prognosis and link them to putative antigen targets, we employed the McPAS and VDJ databases, a curated catalog of previously reported TCRs associated with known pathology-specific antigens [10–12]. Additionally, commonalities among TCRs were analyzed using the Grouping of Lymphocyte Interactions by Paratope Hotspots (GLIPH) algorithm to identify features or motifs of TCRs that may recognize the same antigen [13].

## Materials and methods

### Patient sample collection and processing

Ascitic fluid samples were collected from 57 patients who underwent diagnostic laparoscopy or cytoreductive surgery for a suspected diagnosis of ovarian cancer in the Department of Gynecologic Oncology at The University of Texas MD Anderson Cancer Center. Forty-seven patients were diagnosed with high-grade epithelial ovarian cancer; these patient samples were included in our analysis (Table 1). All patients were enrolled in an Institutional Review Board–approved tumor banking protocol (LAB09-0890), and written informed consent was obtained to collect ascitic fluid at the time of surgery. All patients’ ascitic fluid samples were acquired only once before receiving any treatment. The collected ascitic fluid was centrifuged, and the supernatant fluid was removed; the remaining pellet was resuspended in RPMI 1640 with L-glutamine (MediaTech) and then transferred over Ficoll-Paque Plus Media (GE Healthcare) for gradient separation. The ascitic cell buffy coat was removed and washed with centrifuge steps in RPMI media. Ascitic cells, 10 × 10^6^ cells total per sample, were stored frozen in aliquots of 1 × 10^6^ cells until they were used for flow cytometry or DNA extraction prior to TCR sequencing. Clinical information and survival data were collected from medical records.

**Table 1:** Clinical characteristics of the study cohort (n = 47).

Characteristic	No. (%)
Median age (range)	62 years (45–76 years)
Median body mass index (range)	27.8 kg/m^2^ (19.9–43.5 kg/m^2^)
Median pretreatment CA125 level (range)	770.2 U/mL (26.6–11837.0 U/mL)
Median time from diagnosis to paracentesis (range)	0 days (-4 to 373 days)
Race/ethnicity	
Asian	4 (9)
Black	3 (6)
Hispanic	5 (11)
White	35 (74)
Ascites cytologic findings	
Positive for malignancy	39 (83)
Negative for malignancy	6 (13)
Missing data	2 (4)
Stage	
IIc	1 (2)
IIIa	1 (2)
IIIc	35 (75)
IV	9 (19)
Unstaged	1 (2)
Histologic subtype	
Serous/papillary serous	41 (87)
Mixed mesoderm Mullerian tumor	1 (2)
Mixed	2 (4)
Clear cell	1 (2)
Serous/papillary serous and undifferentiated	1 (2)
Serous/papillary serous and clear cell	1 (2)
Reductive surgery status	
Optimal	38 (81)
Suboptimal	5 (11)
Unresectable	4 (9)
Neoadjuvant chemotherapy	
Yes	32 (68)
No	15 (32)
*TP53* gene mutation	
Positive	10 (21)
Frameshift	1 (2)
Missense	3 (6)
Nonsense	5 (11)
Unknown	1 (2)
Negative	5 (11)
No data	32 (68)
Prognosis	
Worst	4 (9)
Poor	16 (34)
Good	13 (28)
Excellent	14 (30)

### Flow cytometry

One million cells from ascites were washed with a FACS buffer (1× phosphate-buffered saline with 2% fetal bovine serum and 2mM ethylenediaminetetraacetic acid) to be used for immune cell phenotyping. For surface staining, an antibody cocktail was prepared with a 9-color panel following the dilutions recommended by the manufacturer. The panel used for the analysis included antibodies against human CD3 (clone SK7, PE-Cy7) and CD4 (clone RPA-T4, PerCPCy5.5), both from BD Bioscience (San Jose, CA); and CD8a (clone HIT8a, Alexa Fluor 700), PD-1 (clone EH12.2H7, APC-Cy7), and ICOS (clone C.398.4A, FITC), all from Biolegend (San Diego, CA). We excluded dead cells using a LIVE/DEAD fixable dead cells stain kit obtained from Life Technologies (Carlsbad, CA). The cells were incubated with the antibody cocktail at 4°C in the dark for 30 minutes. After the 30-minute incubation, cells were washed twice with FACS buffer and then fixed and permeabilized using the FoxP3 Fix/Perm Kit (ThermoFisher Scientific, Waltham, MA). Next, intracellular staining was performed using the following antibodies: FoxP3 (clone PCH101, Alexa Fluor 488) from eBioscience (Waltham, MA), Ki-67 (clone Ki-67 BV711) from Biolegend (San Diego, CA), and CTLA-4 (clone BN13, BV 786), from BD Bioscience (San Jose, CA). These intracellular antibodies were prepared in a permeabilization buffer and added to the cells, then incubated at 4°C in the dark. Cells were washed with a FACS buffer twice and prepared for collection.

Compensation controls were prepared using OneComp ebeads (cat. no. 01-1111-42; eBioscience, Waltham, MA), and fluorescence-minus-one controls were used. No other positive or negative controls were used. All samples were collected on an LSR Fortessa X-20 analyzer and analyzed using FlowJo software version 10.5.3 (FlowJo, LLC, Ashland, OR). S1 Fig. depicts the gating strategy used to analyze T cells from malignant ovarian ascites. The fluorescence-minus-one controls were used to gate different activation markers expressed on T cells. Sample data available for each patient are shown in S1 Table.

### TCR sequencing

DNA extraction was performed using DNeasy Blood & Tissue Kits (QIAGEN) on one million cells from ascites. Multiplex PCR-based deep sequencing of the CDR3 region of the TCR β-chain, hereafter referred to as CDR3β, was performed using the ImmunoSEQ immune profiling system (Adaptive Biotechnologies, Seattle, WA) [14]. We started with a relatively similar amount of T cells among the groups from one million cells from ascites. The average total productive templates, a predictor of T cell counts, was 48824 (± 27974) in all samples. The multiple comparisons of all the groups (worst, poor, good, and excellent) showed there were no significant differences among groups (all *p* values were > 0.999). We analyzed productive rearrangements, productive entropy, productive clonality, maximum productive frequency, top 10 productive frequency, and top 100 productive frequency. "Productive rearrangements" refers to the number of unique rearrangements in the sample that are in-frame and do not contain a stop codon and produce a functional protein receptor. Samples with higher productive entropy have a greater diversity of rearrangements. The productive frequency is a specific productive rearrangement among all productive rearrangements within a sample. The maximum, top 10, and top 100 productive frequency are the maximum, sum of the top 10, and sum of the top 100 productive frequency values found within a sample. TCR data were available for most patients (S1 Table).

### Statistical methods

#### Survival analysis

Kaplan-Meier analysis was used to determine the ability of immune and clinical characteristics to predict OS, defined as the time from diagnosis to death from any disease-related cause. The Kaplan-Meier method was implemented using R libraries "survival" and "survminer." Optimal cutoff points for continuous variables were calculated using function "surv_cutpoint" from the "maxstat" R package. Categorization of the variables according to the cutoff points was done using function "surv_categorize." Cox proportional hazards models [15] were used to analyze the impact of immune and clinical characteristics on OS. The models were produced by function"coxhp" from the R library "survival."

#### Flow cytometry data analysis

The abundance values of immune markers, expressed as a fraction of CD3+ live lymphocytes, were log_2_ transformed, normalized, and hierarchically clustered using open source clustering software with default parameters [16]. The number of clusters was evaluated using the "gap_stat" algorithm [17] implemented in the function fviz_nbclust() from the "factoextra" R library. A heat map was generated using Java TreeView 1.1.6 [18]. Association of the immune markers with prognosis was evaluated using the Kruskal-Wallis rank-sum test followed by the Dunn test using R library"dunn.test." Visualization of the associations was done in R using the "boxplot" function and library "beeswarm."

#### TCR sequencing data analysis

TCR sequencing analysis was initially implemented using the immunoSEQ Analyzer 3.0 to preprocess the data and obtain sample-level statistics, including total/unique counts and rearrangements for all sequences, productive rearrangements, entropy, clonality, and maximum frequency. Pairwise association of the TCR characteristics and flow cytometry immune marker abundance values was tested using Pearson correlation after log_2_ transformation of the parameters. Association of the characteristics with OS was evaluated using the Kruskal-Wallis rank-sum test. This test, combined with the Fisher test, was also used to evaluate the association of each CDR3 peptide found in the cohort with prognosis. Two external -immune receptor databases, McPAS-TCR [10] and VDJdb [11] were used to annotate clonotypes derived in the study. TCRs associated with prognosis according to the tests (unadjusted p < 0.05) were annotated by McPAS [10] using the Levenshtein distance threshold to be equal to 1. A similar annotation was done for all TCRs found in the cohort. The produced tables with annotations were compared with all annotations deposited in McPAS (April 14, 2020). For this comparison, we quantified functional categories (pathogens, cancer, autoimmune, allergy, other) using the number of annotations that belonged to each category in each annotation table and in the database. The chi-square goodness-of-fit test was used to compare the observed distribution of the number of annotations in terms of the categories with the expected probability distribution in the database. Annotations of TCR sequences in the cohort by McPAS pathologic condition were also searched by their association with prognosis. Then, the normalized number of annotation records that belonged to each pathologic condition was compared among patients in 4 groups (worst prognosis, good prognosis, poor prognosis, and excellent prognosis) using the Kruskal-Wallis test. In case of VDJdb annotation [11], the cancer associated records were downloaded from the database on 3 Nov 22 and filtered to leave only CDR3β complementary region and human epitopes. Only annotations with a complete match of CDR3β peptides between the database records and the study cohort were considered. Match of the segments, Variable (*V*), Diversity (*D*), and Joining (*J*), were not required; although further calculation of their usage in each patient of the study cohort was done for records annotated by cancer associated CDR3β.

#### TCR grouping

The TCRs found in the cohort were also used for clustering in GLIPH software [13] to predict the putative binding of the TCR to the same MHC-restricted peptide antigen and to find CDR3 motifs enriched in each cluster relative to the unselected TCRs. For the clustering, we selected CDR3β peptides covered by more than 1 read. GLIPH software was used with default parameters, and two reference files of T lymphocyte CDR3β peptides, CD4 and CD8, were used to search for clusters. Each inferred cluster with at least 4 subjects (10% condition rule in statistics) was analyzed using chi-square contingency tables to evaluate the association of the cluster with excellent prognosis and poor or worst prognosis. For cluster association with an excellent prognosis, the contingency table was created for each cluster by calculating the number of TCRs annotated by excellent prognosis (group 1) and the rest (group 2: worst, poor, and good prognosis) in the cluster and the dataset. For cluster association with poor or worst prognosis, we compared the number of TCRs annotated by worst and poor prognosis (group 1) with those annotated by the remaining prognoses (group 2: excellent and good prognosis). We considered a cluster associated with prognosis if the p-value of group 1, when compared with group 2, was less than 0.05. For example, a TCR cluster enriched with excellent prognosis values that do not include any worst or poor prognosis values would be associated with an excellent prognosis. A cluster enriched with worst or poor prognosis values that do not include any excellent prognosis values would be associated with poor/worst prognosis. The clustered CDR3β peptides were aligned, and then the phylogenetic tree was generated by Clustal Omega 1.2.4, using the Neighbour-joining algorithm without distance corrections [19]. The sequence logo corresponding to the alignment was generated by Seq2Logo-2.0 [20].

Association network analysis [21] was used to validate the statistical approach underlying the selection of GLIPH clusters linked to excellent prognosis or poor/worst prognosis. In that analysis, we considered samples combined in the GLIPH cluster to be associated, and we generated a list of the samples for each GLIPH cluster of peptides linked to poor/worst or excellent prognosis. The lists were used as input for the association network software [22] to generate a network of the associated samples. Two separate networks were generated for CD4 and CD8 reference using the default parameters. Pearson correlation was used to measure pairwise similarity between samples, with a correlation coefficient threshold of R = 0.60. Cytoscape [23] was used to visualize the networks.

## Results

### Patient characteristics

The clinical characteristics of all 47 patients in our analysis are shown in Table 1 and S1 Table in more detail. Ascitic fluid was collected from patients suspected of having ovarian cancer who underwent laparoscopy for evaluation and diagnosis. Eighty-three percent of patients had malignant ascites. Ninety-four percent of patients had advanced-stage (IIIC or IV) disease, 91% had serous histologic characteristics, and all had high-grade ovarian cancer.

Patients were classified according to their response to therapy, including surgery and chemotherapy (adjuvant or neoadjuvant), into 2 groups: those with recurrence-free survival (RFS) less than 6 months and those with RFS longer than 6 months. Each of these groups was then further subdivided into 2 subgroups. For those with RFS <6 months, one subgroup included patients whose disease did not respond to primary treatment or had an incomplete response by the end of the first-line therapy; this group is hereafter referred to as the worst prognosis group. Those with RFS <6 months whose disease responded to treatment are hereafter referred to as the poor prognosis group. Those with RFS >6 months and those with RFS up to 12 months are hereafter referred to as the good prognosis group. The remaining patients with RFS >12 months; these patients are hereafter referred to as the excellent prognosis group.

### Association of patient prognosis with immune cell populations and TCR characteristics

**Fig 1:**
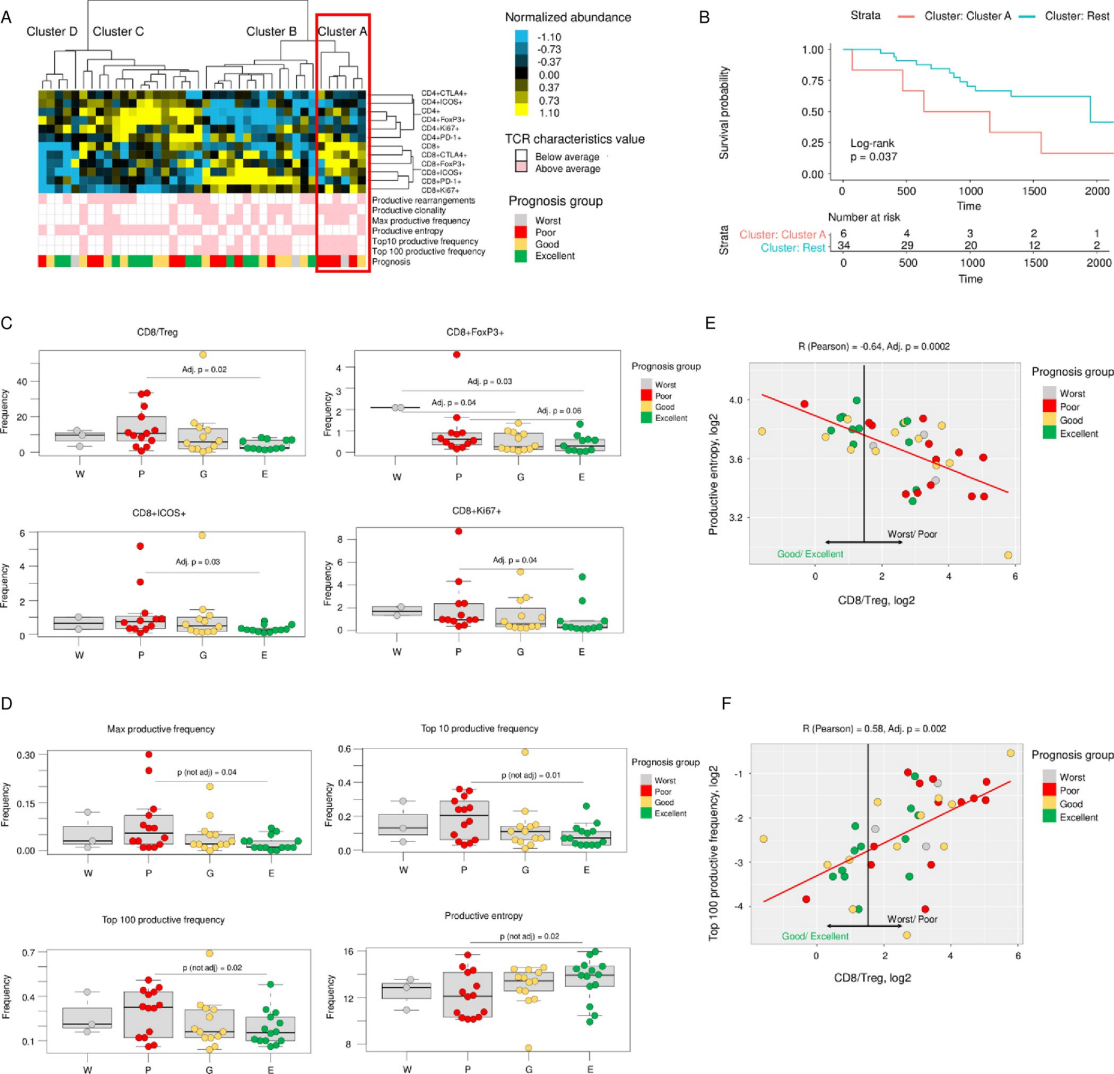
Association of immune characteristics determined by flow cytometry and T cell receptor (TCR) sequencing with patient prognosis. (A) Unsupervised hierarchical clustering of samples (columns) in terms of the profile of immune marker abundance determined by flow cytometry. Cell color in the heatmap shows normalized abundance of immune markers, from low (blue) to high (yellow). Pink and white boxes show values of TCR characteristics; pink indicates an above-average value and white indicates a below-average value for each characteristic: productive rearrangement, productive clonality, productive entropy, top 10 productive frequency, and top 100 productive frequency. Cluster A (red square) is enriched with patients with a poor or worst prognosis; no patients in this cluster had an excellent prognosis. (B) Kaplan–Meier curves of overall survival in patients grouped in Cluster A and in the remaining clusters by hierarchical clustering. (C) CD8+ T cell subpopulations were significantly decreased in ascites from patients with an excellent prognosis compared with ascites from patients with a poor prognosis. The y-axis shows the percentage of CD3+ cells (log_2_). CD8/Treg is the ratio of the percentage of live CD8+ lymphocytes to the percentage of live CD4+FoxP3+ lymphocytes. Each box plot represents the median and the twenty-fifth and seventy-fifth percentiles. Only significant associations are shown. (D) Significant differences in ascites TCR characteristics between patients with excellent and poor prognosis. The y-axis shows the percentage of CD3+ cells (log_2_). (E) Scatterplots of CD8/Treg with productive entropy. (F) Scatterplots of CD8/Treg with top 100 productive frequency. Good/excellent prognosis was associated with less abundant CD8/Treg, increased productive entropy, and decreased top 100 productive frequency.

We analyzed frequencies of CD4+ and CD8+ T cells and their subpopulations with expressed markers of activation and inhibition using flow cytometry, and we investigated the association of these cell subpopulations, as well as TCR characteristics, with prognosis. Unsupervised hierarchical clustering of the immune markers (Fig 1A) identified 4 groups by flow cytometry immune marker abundance. One group (referred to as Cluster A) was notably lacking patients with an excellent prognosis. The OS of patients in Cluster A was the worst among the groups (Fig 1B). The other groups (Clusters B, C, and D) did not show significant associations with prognosis.

Cluster A ’patients’ immune environment was characterized by a significant increase in the abundance of CD8+ T cells (p = 0.001) and a decrease in the abundance of the CD4+ICOS+ activation marker (p = 0.02) compared with the other clusters (S2 Fig). The abundance of CD4+ and CD4+FoxP3+ T cells, as well as the CD4/CD8 ratio, was also significantly reduced in Cluster A (p = 0.05, p = 0.002, and p = 0.005, respectively). As a result, the ratio of CD8+ T cells to regulatory T cells (CD8/Treg), calculated as a ratio of CD8+ to CD4+FoxP3+ cells, was significantly higher in Cluster A (p = 0.0003) than in the rest of the clusters (S2 Fig).

Several TCR characteristics were significantly increased in Cluster A patients, including productive clonality (p = 0.02), maximum productive frequency (p = 0.02), top 10 productive frequency (p = 0.02), and top 100 productive frequency (p = 0.04; S3 Fig).

The observed associations were confirmed by statistical testing of each flow cytometry immune marker and TCR characteristic (S2 Table) using the 4 prognosis groups. We observed significant decreases in the CD8/Treg ratio and reduced frequencies of CD8+FoxP3+, CD8+ICOS+, CD8+Ki67+, and CD8+CTLA-4+ cells (Fig 1C, S2 Table) in patients with an excellent prognosis compared with those with a poor prognosis. For TCR characteristics, including maximum productive frequency, top 10 productive frequency, top 100 productive frequency, and productive clonality were decreased in patients with an excellent prognosis (Fig 1D, S2 Table). Productive entropy was increased in those with an excellent prognosis. This pattern reveals more restricted T cell repertoire or a more clonal TCR architecture in ascites of patients with poor prognoses.

Analysis of pairwise correlations between TCR characteristics and immune subpopulation determined by flow cytometry identified a significant CD8/Treg ratio association with productive entropy and productive frequency (S3 Table). Specifically, the proportion of CD8+ T cells to regulatory T cells in ascites correlated negatively with productive entropy (p = 0.0002; Fig 1E) and positively with top 100 productive frequency (p = 0.002; Fig 1F). In summary, ascitic fluid from patients with an excellent or good prognosis had a low CD8/Treg ratio with high productive entropy and low productive frequency of the TCR repertoire.

### Association of clinical and immune characteristics with OS

**Fig 2:**
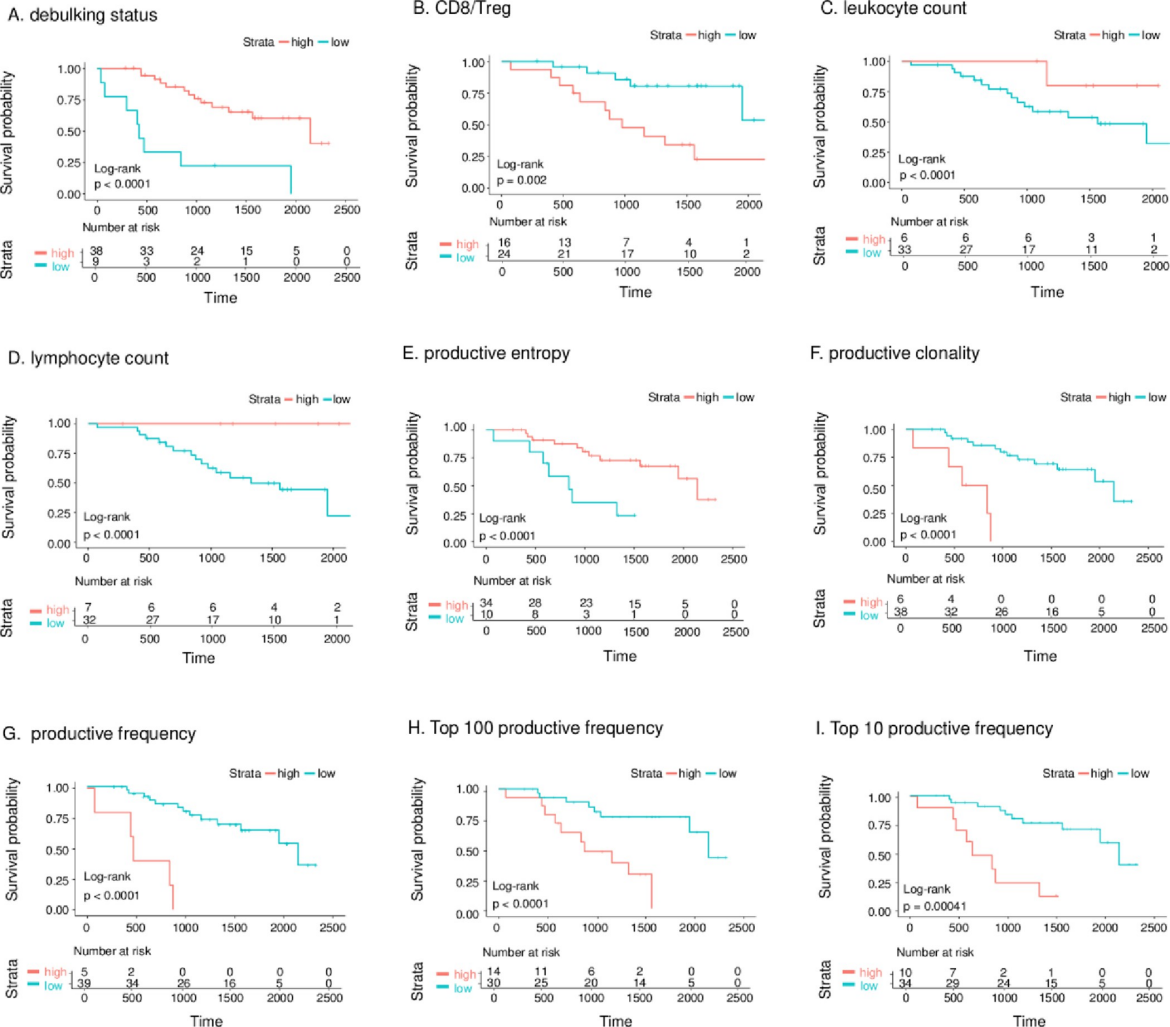
Kaplan–Meier curves of overall survival for various factors. (A) Debulking status. (B) CD8/Treg ratio. (C) Total leukocyte counts. (D) Total lymphocyte count. (E) Productive entropy. (F) Productive clonality. (G) Maximum productive frequency. (H) Top 10 productive frequency. (I) Top 100 productive frequency.

Immune characteristics identified by TCR sequencing and flow cytometry were also evaluated as potential biomarkers of OS. Because some clinical characteristics have known effects on OS, these were also assessed as potential confounding factors. To find the strong confounders, we collected routine clinical information, including debulking status, type of the chemotherapy (adjuvant or neoadjuvant), stage, race/ethnicity, body mass index, and histologic subtype (Table 1). Then, we evaluated the prognostic value and confounding effects of these variables using the Kaplan-Meier method and the Cox proportional hazards model. Among the clinical characteristics, debulking surgery had the most significant positive association with OS (unadjusted log-rank p < 0.001; Fig 2A), indicating that this variable had a strong confounding effect.

Of all the immune characteristics studied by flow cytometry, only CD8/Treg was significantly associated with OS after the adjustment for multivariable testing (adjusted log-rank p = 0.04; Fig 2B). According to the univariate regression model, CD8/Treg was a good prognostic factor of OS, with a likelihood ratio test p-value of 0.01 and hazard ratio of 1.69 (S4 Table). Adjustment of the model for debulking status showed that the CD8/Treg had a significant independent effect on OS. In the multivariable model with CD8/Treg and debulking status as covariates, the likelihood ratio test p value was decreased to 7e-04, and the hazard ratio of CD8/Treg was increased 1.995 (S4 Table). OS was also associated with total leukocyte and lymphocyte counts in ascites, as determined by flow cytometry (Fig 2C and 2D), which may be a result of the cell viability bias; patients with better OS had a more significant percentage of cells recovered after the thawing and staining process (S4 Fig).

TCR characteristics, including productive clonality, maximum productive frequency, productive entropy, top 10 productive frequency, and top 100 productive frequency (Fig 2E–2I), were also associated with OS (log-rank p values adjusted for multivariable testing ranged from 0.01 to 4.47e-06). According to the multivariable analysis, each characteristic was a good predictor of OS independent of debulking status (S4 Table). The overall significance of the models evaluated by the likelihood ratio test was less than 0.001.

### Association of TCR functional categories with prognosis

**Fig 3:**
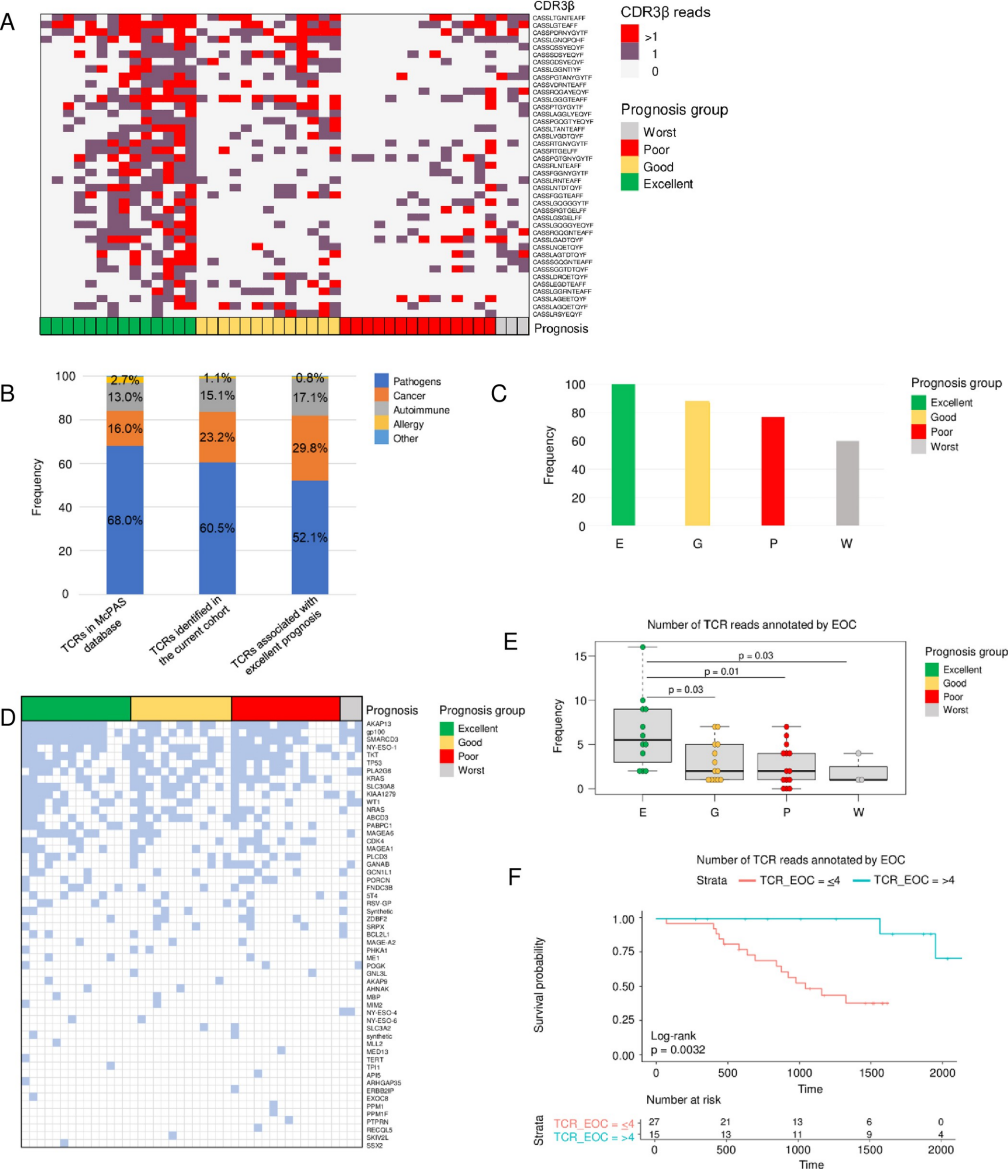
Association of CDR3β peptides with prognosis and annotation in McPAS and VDJ databases. (A) Heatmap of the CDR3β peptides associated with prognosis in the current study, showing that the identified CDR3β peptides are more common and abundant in patients with an excellent prognosis. (B) Annotation by McPAS functional category of CDR3β peptides found in all T cell receptors (TCRs) annotated in the McPAS database (left), TCRs found in the current cohort (middle), and TCRs associated with prognosis in the current cohort (right). The annotation revealed that CDR3β peptides associated with excellent prognosis are enriched along with those activated in cancer (orange) and depleted along with those activated by pathogens (blue) in the current cohort compared with McPAS data. (C) Frequency of cancer associated CDR3β annotated by VDJdb in patients with different prognosis. Frequency was calculated for each prognosis group as number of CDR3β matched cancer associated CDR3β from VDJdb divided by number of patients in the group. (D) Cancer associated epitope genes found in the study cohort by complete match of the respective CDR3β in VDJdb. Epitope genes are in decreasing order by number of patients, and patients in each prognosis group are ordered by number of epitope genes. Epitope genes, such as MLANA, IGF2BP2, BST2, SEC24A, SF3B1, and PMEL, found in more than 75% patients, were excluded. (E) CDR3β peptides annotated by McPAS as neoantigens activated by epithelial ovarian cancer (EOC) were more abundant in patients with an excellent prognosis. (F) Overall survival of 19 patients with low TCR gene rearrangement and 20 patients with a high number of reads annotated by EOC TCR reads. A TCR was attributed to a Kaplan-Meier estimate with a 95% confidence interval. The two-sided log-rank test was used to calculate *p* values.

The TCR repertoire in ascites was further evaluated to identify TCR peptides enriched in patients with an excellent prognosis. Forty-one individual CDR3β peptides associated with the 4 prognosis groups were identified (Fig 3A). Functional annotation of the peptides based on relationships with known pathologic conditions was done using the McPAS database, a manually curated catalog of pathologic conditions associated with TCR sequences from previously reported studies [10]. The McPAS-TCR database has TCR sequences annotated by 4 major categories: pathogens (68%), cancer (16%), autoimmune (13%), and allergy (2.7%; Fig 3B, left bar). Annotation of peptides identified in ascites in the current study cohort (Fig 3B, middle bar) revealed a significant increase in peptides recognizing cancer antigens (an increase from 16% to 23.2%) and a decrease in peptides recognizing pathogens (a reduction from 68% to 60.5%). The difference was significant (p < 0.003) according to the chi-square goodness-of-fit test. The percentage of TCRs predicted to recognize cancer antigens was even higher (29.8%) for peptides associated with excellent prognosis (chi-square goodness-of-fit test p < 0.001; Fig 3B, right bar), revealing a potential link between peptides recognizing cancer antigens in ascites and excellent prognosis.

Increased number of cancer associated antigens in ascites of patients with excellent prognosis was confirmed by annotation of the TCR repertoire in the study cohort using VDJdb [12]. Frequency of CDR3β with complete match to peptides that bind known cancer neoantigens from VDJdb was 87% and 45% greater in a patient with excellent prognosis than worst or poor, respectively (Fig 3C). Many known oncogenes, such as TP53, AKAP13, SMARCD3, TKT, NY-ESO-1, KRAS, and NRAS, frequently mutated in ovarian and other solid tumors are found among the annotated epitope genes, revealing surprisingly immunogenic environment of ascites in patients with excellent prognosis (Fig 3D).

### Increased abundance of TCRs recognizing ovarian cancer antigens in ascites of patients with an excellent prognosis

Annotations of TCR sequences in the cohort by pathologic condition according to McPAS revealed 33 pathologic conditions (S5 Table). Among them, only epithelial ovarian cancer was associated with prognosis (Kruskal-Wallis p = 0.03). Patients in the excellent prognosis group had significantly more epithelial ovarian cancer–associated TCRs than did patients in the worst prognosis group (p = 0.003), poor prognosis group (p = 0.04), or good prognosis group (p = 0.04; Fig 3E). OS was also significantly better (p = 0.01) for patients with more abundant (greater than the median) epithelial ovarian cancer–associated peptides in ascites (Fig 3F). The most abundant epithelial ovarian cancer TCRs were annotated by a known neoantigen peptide of the transcription factor *TP53*, the most commonly mutated gene in HGSOC [24]. Given that similar but not identical TCRs can recognize the same antigen, we looked for a common motif within the TCRs that may bind the same neoantigen. To accomplish this, we used GLIPH software [13]. Using the CD4+ T cell reference files, we identified a cluster of CDR3β peptides (S5 Fig) that includes the known CDR3β peptide (CASSLVNTEAFF) annotated by epithelial ovarian cancer and the *TP53* neoantigen in McPAS. The common pattern of the peptides (CA%SL%NTEAFF) was found in 22 patients with various prognoses, including 4 patients with the peptide recognizing the known *TP53* epitope (RCSDSDGLAPPQNLIRVEGNLRVEY; S6 Table). Interestingly, the same CDR3β peptide (CASSLVNTEAFF) that binds known *TP53* epitope was found in patients that have different variable (V) segments, and the same, TCRBJ01-01*01, joining (J) segments that may indicate convergent recombination and potential cross reactivity of the TCR clones. Two V segments, TCRBV11-02*02 and TCRBV07-08*01, originated CASSLVNTEAFF CDR3β more frequently in 32% of the studied patients (S7 Fig). Overall, however, patients with excellent and poor prognoses in the study did not significantly differ in V/J gene segment usage.

### Mutation status of TP53 and BRCA1/BRCA2 in tumors and TCR characteristics in ascites

Upon observing a potential link between peptides that recognize cancer neoantigens in ascites and better prognosis, we examined the mutation status of 3 genes that are frequently altered in ovarian tumors: two homologous recombination repair genes (*BRCA1* and *BRCA2*) and the transcription factor *TP53* (S1 Table). We evaluated the association of mutation status with TCR characteristics in ascites and with OS. *BRCA1/2* mutations were evaluated in 32 patients, and mutations were found in 7 cases. In this small subgroup, *BRCA1/2* mutations were often accompanied by productive rearrangements in ascites (one-sided Mann-Whitney p = 0.07; S6 Fig), which is consistent with reports that *BRCA1/2*-deficient cancers have an increased burden of mutations and neoantigens [25, 26]. However, no significant association was found between *BRCA1/2* mutation status and OS in the study cohort.

We then determined whether *TP53* gene mutations and p53 transcription factor abundance in tumors were associated with CDR3β peptides annotated by the CA%SL%NTEAFF pattern in ascites and with OS. The presence of *TP53* mutations was evaluated in 15 patients, and 10 of them had *TP53* mutations (S1 Table). The abundance of p53 transcription factors was also evaluated by immunohistochemistry in 20 tumors; 14 of them were p53-positive (wild-type). There were 8 samples with both *TP53* and p53 data available, and these samples showed perfect consistency. Four p53-positive (wild-type) tumors did not have *TP53* mutations, and four p53-negative tumors had *TP53* mutations (S1 Table). Of the 4 samples that did not have *TP53* mutations, three had TCRs with the CA%SL%NTEAFF pattern, and only one had TCRs without the pattern (S6 Table). *TP53* mutation status and p53 abundance were not associated with the putative TCR receptors that bind *TP53* epitopes or OS, likely because of the small size of the cohort.

### TCR peptides potentially associated with prognosis

**Fig 4:**
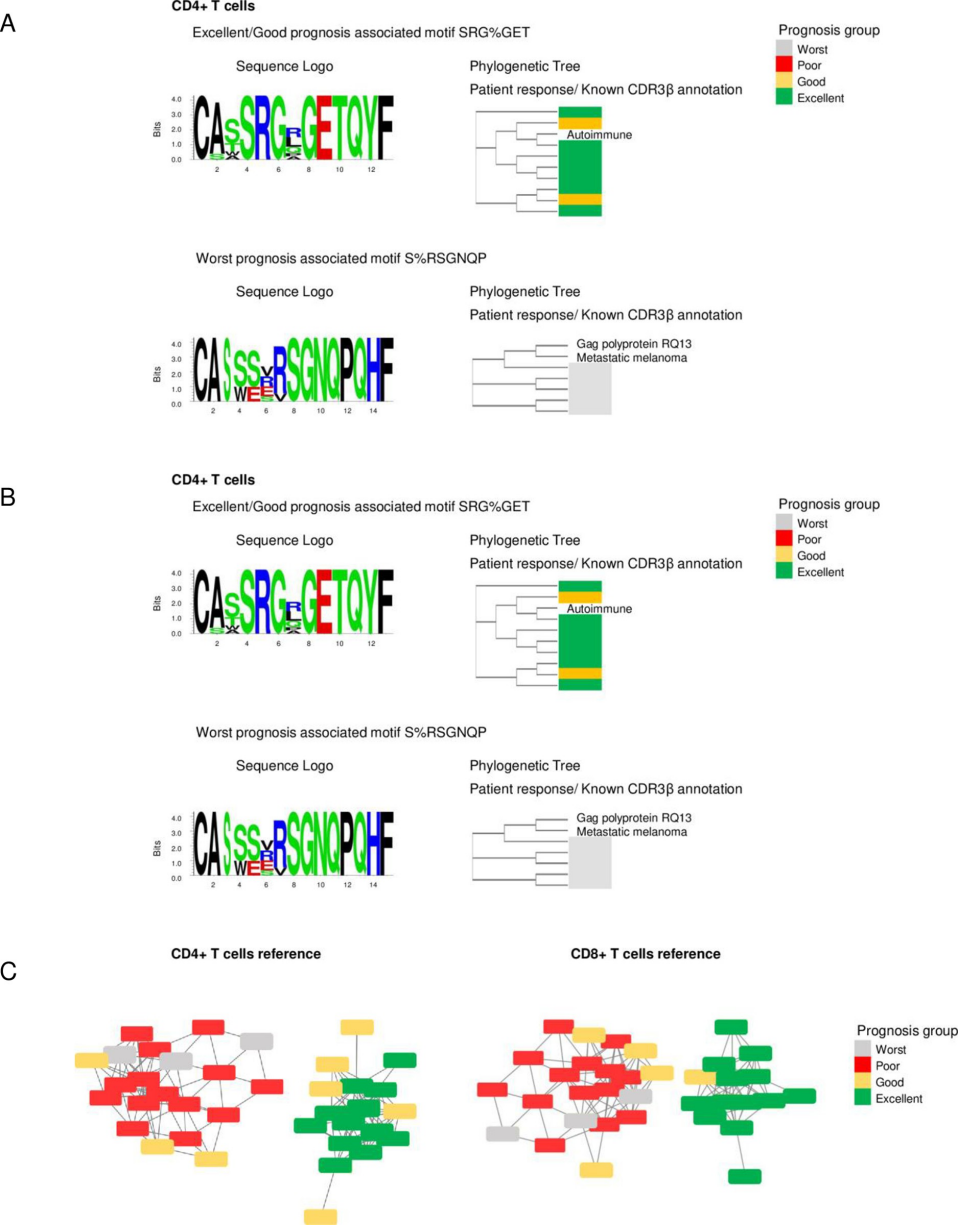
Ascitic fluid cell CDR3β motifs associated with prognosis. (A) Examples of CD4+ T cell receptor (TCR) motifs associated with different prognoses. (B) Examples of CD8+ TCR motifs associated with different prognoses. The figures show the logo and phylogenetic tree of the clustered CDR3β peptides for each motif. The phylogenetic tree also includes peptides found as the best hit from McPAS; the complete set of motifs associated with excellent or poor/worst prognosis is shown in S7A Table. (C) Association networks constructed to form the cluster of motifs associated with response to platinum therapy. Nodes in the networks indicate individual patients; the color of the node indicates prognosis. Edges in the networks indicate an association between a pair of samples if they share GLIPH specificity groups associated with prognosis. The networks validated the statistical algorithm used to identify prognosis-associated specificity groups.

We next analyzed the complete set of predicted CD4+ and CD8+ T cell CDR3β motifs to find those associated with patient prognosis (Fig 4A–4C, S7 Table).

In CD4+ T cells, we found 98 clusters (392 unique CDR3β motifs) associated with excellent prognosis and 133 clusters (542 unique CDR3β motifs) associated with worst prognosis. In CD8+ T cells, we found 48 clusters (237 unique CDR3β) related to excellent prognosis and only 14 clusters (121 unique CDR3β) associated with worst prognosis. Fig 4A and 4B provide specific examples of the identified motifs of T-helper cells (CD4+ T cells) and cytotoxic T cells (CD8+ T cells). For each T cell type, we identified motifs associated with prognosis.

To validate the association of the selected TCR clusters with prognosis, we generated an association network of samples, in which samples combined in each cluster with a shared motif pattern were considered associated (Fig 4C). Two samples in the network were connected if they had similar profiles composed of the shared patterns between each pair of samples. Both networks, CD8+ T cells and CD4+ T cells, showed a clear separation of samples by prognosis, thus validating the algorithm underlying the selection. Further annotation of the selected unique CDR3β motifs using McPAS-TCR identified several putative neoantigens that can be recognized by the selected peptides (S7A Table). Among the neoantigens are well-known genes associated with tumor progression and metastases, such as *HAUS3* [27], *NSDHL* [28], *MAGEA10* [29], *FNDC3B* [30], *TEAD1* [31, 32], *DHX33* [33], *PGM5* [34], and *MLL2* [35]. These results indicate that the ascitic fluid environment is very complex, and ascitic fluid enriched with TCRs that recognize epithelial ovarian cancer neoantigens may be associated with improved patient outcomes.

## Discussion

Ascitic fluid composition, both cellular and acellular, is thought to support the intraperitoneal spread of ovarian cancers [36, 37]. Although HGSOC is initially chemosensitive, owing to highly proliferative activity and defects in DNA repair capacity, chemoresistance almost always emerges [3], and the tumor typically metastasizes within the peritoneum. To determine the role of immune characteristics of ascites in the development of ovarian cancer resistance to platinum-based chemotherapy, we grouped ovarian cancer patients who received standard platinum-based chemotherapy according to RFS, reflecting the time to relapse and chemotherapy response. Our results indicate that T cell phenotype, including the antigen specificity of T cells, in the ascites of ovarian cancer patients may be a determinant of OS and platinum sensitivity.

Prior studies have reported that a higher absolute number of CD8+ T cells is associated with improved OS rates in ovarian cancer [38]. However, in the current study, we observed a higher percentage of CD8+ T cells in Cluster A (S2 Fig), and OS was the worst in this group (Fig 1B). Analysis of CD8+ T cell subpopulations revealed heterogeneity and significant positive association of some subpopulations, such as CTLA-4+, FoxP3+, ICOS+, and Ki67+, with excellent prognosis rather than poor prognosis (S2 Table). Although further validation is needed for poly-functional CD8+ T cells expressions by looking into activation markers such as HLA-DR and CD69, the strongest positive association was observed for CD8/Treg, which was also associated with TCR characteristics, including top 100 productive frequency and productive entropy (Fig 1E and 1F). This relationship suggests an outgrowth of some TCR clones in patients with poor prognoses. Ascites-derived T cells from ovarian cancer patients have been reported to frequently express co-inhibitory receptors [39], which can lead to T cell dysfunction in ovarian cancer [40], and CD4+ T cells expressing the transcription factor FoxP3 usually favor tumor progression [41–43]. However, this correlation is highly variable and depends on the tumor type [44]. More recent data suggest that in certain cancers characterized by chronic inflammation, such as colon, breast, bladder, or head and neck cancer, intra-tumor accumulations of T regulatory cells appear to be associated with favorable prognosis and improved OS [45, 46]. An inflammatory environment rich with inflammatory cytokines, growth factors, and chemokines is well-documented in ascites [47–49]. A higher level of the pro-inflammatory factor Cyr61 was also reported in ovarian cancer ascites when compared with the tumor itself [50].

Although validation is needed from analyses such as Luminex-based cytokine/chemokine levels in ascites, the elevated expression of regulatory T cells (CD4+FoxP3+) in patients with a good prognosis (Clusters B, C, and D; S2 Fig) may indicate that these cells play a role in suppressing tumor-promoting inflammation. In a recent study of ovarian cancer ascites using multiplex bead-based immunoassays, high concentrations of 2 common inflammatory factors, matrix metalloproteinase 2 and 3, were identified as predictors of shorter platinum-free intervals in patients treated with neoadjuvant chemotherapy or primary debulking surgery [51]. Consistent with the observation, we find that low CD8/Treg in ascites could be a good marker of favorable prognosis in ovarian cancer, along with debulking surgery and TCR characteristics, including high number of productive rearrangements and productive entropy, low productive clonality, maximum productive frequency, top 10 productive frequency, and top 100 productive frequency.

TCR sequencing in ascites identified CDR3β peptides associated with good prognosis and epitopes that could have utility as targets of neoantigen vaccines. We found that patients with a higher number of reads mapped to known epithelial ovarian cancer TCR gene rearrangements in malignant ascites had longer OS (Fig 3F). In particular, the CDR3β chain rearrangement CASSLVNTEAFF, observed in the good prognosis group, recognizes the mutant *TP53* neoantigen peptide, CSDSDGLAPPQNLIRVEGNLRVEY (S5 Fig). TCR clustering predicted that almost half of the studied patients had TCRs that could recognize the same *TP53* epitope. However, these receptors can recognize not only this specific epitope but also other epitopes because of TCR cross-reactivity, an ability to recognize more than one epitope, which is well documented [52]. In fact, the same CDR3 peptide CASSLVNTEAFF was used by the GLIPH software in a training set of TCRs of known specificity [53]. The peptide was initially annotated by the ability to bind epitope LPRRSGAAGA, studied as part of the nucleoprotein from influenza A virus (ID 38688 in The Immune Epitope Database) [54]. This suggests that CDR3β chain rearrangement recognizing *TP53* may have initially evolved to fight influenza and later played a role in the immune surveillance of malignant cells with *TP53* mutations. In addition, usage of different V gene segments in the TCR sequence and presence of the sequence in different patients suggest that the TCR clone may represent a preferential assembly of VDJ segments [12]. The observation makes it a good target for ovarian cancer vaccine development.

Consistent with this observation, in our study, of the 4 patients with p53-positive (wild-type) tumors without *TP53* mutations, three had TCRs with the CA%SL%NTEAFF pattern, and among the 2 patients with p53-negative tumors with *TP53* mutations, none had TCRs without the pattern (S1 and S6 Tables). Pathogenic *TP53* mutations are identified in ~97% of HGSOC, and those mutations are usually found in specific locations, exons 4–7 [55, 56]. Our finding provides a biological rationale for targeting *TP53* neoantigens by developing vaccines. Experimental studies must be specifically designed to explore this opportunity.

In addition to the TCR specificity group linked to the binding of the *TP53* epitope in ascites, we identified several other groups associated with good or poor prognosis. Further annotation of the clustered peptides revealed some putative epitopes targeted by the clusters. We found neoantigens of several genes among the targets with a well-documented association with tumor progression and metastasis. This approach may be beneficial for discovering epitope-TCR pairs associated with a specific phenotype. One such example is a TCR specificity group identified in our study that may recognize a *TEAD1* neoantigen. The *TEAD1* gene is a co-activator of YAP, and both are required to maintain stemness and chemoresistance in ovarian cancer-initiating cells [32, 57]. CDR3β peptides identified in the specificity group can recognize cells with the *TEAD1* neoantigen; therefore, CD8+ cells in patients harboring the receptors can target ovarian cancer-initiating cells for destruction, reducing chemoresistance and the probability of recurrence. Indeed, TCRs clustered in this group were found only in patients with excellent prognoses (Fig 4B). Considering that the *TEAD1* epitope recognizing peptides was found only in patients with an excellent prognosis and a high percentage (43%), peptide vaccines may also be a promising strategy to create more durable responses to conventional treatment. Tumor sequencing is needed to confirm the presence of these mutations and functionally demonstrate the binding characteristics of the identified peptides and cognate TCRs to definitively establish these proposed links between TCR motifs and candidate neoantigens. Ultimately, adoptive cell therapy or vaccine approaches with platinum-based chemotherapy may increase the probability of sustained responses and the number of responders [58].

The current study has some limitations. First, we had limited cases where ascites were negative for malignancy. The groups of positive and negative malignancies were unbalanced, thus underpowered to detect differences between patients with and without cancer. One important limitation is the relatively small number of patients in the cohort and incomplete information about their survival outcomes. This did not allow us to evaluate the effects of a combination of factors on prognosis and consider the interactions of these factors. Further studies in a larger cohort of patients are necessary to validate the prognostic value of the immune characteristics in ascites we identified.

Another important limitation of the study was using frozen cells instead of fresh cells for flow cytometry. Recovery of the frozen cells introduces a bias due to differences in cell viability, which may affect more vulnerable cell populations. Therefore, the current study could not measure the abundance of cell populations in absolute counts. This quantification would be very important for comparison with HGSOC tumors, where CD8+ T cell infiltration is a key prognostic factor [7]. Most relationships discovered in the current study, such as associations of TCR characteristics and specific CDR3β peptides with prognosis, are only computational predictions. Further experiments are needed to demonstrate functional links and determine the therapeutic utility of the predictions.

## Supporting information

S1 Fig Gating strategy for the detection of different immune cell subsets from malignant ascites of ovarian cancer patients. Cells collected from ascites were stained with a 9-color flow cytometry panel and analyzed in an X-20 Fortessa flow cytometer. (A) The gating strategy is depicted starting with the detection of lymphocytes, followed by live cells and CD3+ T cells that are separated according to CD4 and CD8 expression. (B) Fluorescence-minus-one (FMO) staining is shown for the different markers analyzed on CD4+ and CD8+ T cells.
(PDF)

S2 Fig Difference in flow subset (expressed as a percentage of CD3+ live lymphocytes) between ascites of patients grouped in Cluster A and ascites of patients grouped in the remaining clusters (others). (PDF)

S3 Fig Difference in T cell receptor (TCR) characteristics between ascites of patients grouped in Cluster A and ascites of patients grouped in the remaining clusters (others). (PDF)

S4 Fig Significant increase in overall survival in patients with a high percentage of ascitic fluid cells recovered after the thawing and staining process. (PDF)

S5 Fig Sequence logo and phylogenetic tree of 41 CDR3β peptides identified by GLIPH (CD4 reference) as a specificity group with a known peptide (CASSLVNTEAFF) that binds the TP53 neoantigen in EOC. (PDF)

S6 Fig Increased T cell receptor productive rearrangements in ascites of patients with *BRCA1/2* tumor mutations. (PDF)

S7 Fig Usage of V gene segments originated known CDR3β peptide recognizing TP53 epitope in 22 patients and, in comparison, the usage calculated for all CDR3β peptides in the cohort. (PDF)

S1 Table TCR sequencing and T cell immune activity according to flow cytometry data for each patient, including p53, *TP53* mutation, *BRCA* mutation, and HRD status. X indicates that data were obtained, and blank indicates missing data. Abbreviations: TCR, T cell receptor; FLOW, flow cytometry; HRD, homologous recombination deficiency; IHC, immunohistochemistry; ND, no data; SNV, single-nucleotide variant.
(PDF)

S2 Table Association of CD8+ T cell subsets and T cell receptor (TCR) characteristics with different prognoses using the Kruskal-Wallis rank-sum test and Dunn test. (PDF)

S3 Table Pairwise association between T cell receptor (TCR) characteristics and T cell subsets identified by flow cytometry, using the Pearson correlation test (no adjustment). (PDF)

S4 Table Cox proportional hazards models of flow cytometry and T cell receptor characteristics with a prognostic value independent of debulking status. Significance is labeled as *<0.05, **<0.01, ***<0.001.
(PDF)

S5 Table Association of pathologic conditions inferred from McPAS annotation with different prognoses according to the Kruskal-Wallis rank-sum test and Dunn test. (PDF)

S6 Table Productive T cell receptor rearrangements combined by the GLIPH algorithm in the specificity group with a known CDR3β peptide binding a *TP53* neoantigen. Red text indicated known CDR3β peptide (CASSLVNTEAFF) annotated by epithelial ovarian cancer and the *TP53* neoantigen in McPAS.
(PDF)

S7 Table Neoantigens found by **A**, McPAS and **B**, VDJdb annotation among clustered CDR3β peptides associated with excellent or poor/worst prognosis.
(PDF)
