# Correction: Immune environment and antigen specificity of the T cell receptor repertoire of malignant ascites in ovarian cancer

**DOI:** 10.1371/journal.pone.0314643

**Published:** 2024-11-22

**Authors:** Kyoko Yoshida-Court, Tatiana V. Karpinets, Aparna Mitra, Travis N. Solley, Stephanie Dorta-Estremera, Travis T. Sims, Andrea Y. Delgado Medrano, Molly B. El Alam, Mustapha Ahmed-Kaddar, Erica J. Lynn, K. Jagannadha Sastry, Jianhua Zhang, Andrew Futreal, Alpa Nick, Karen Lu, Lauren E. Colbert, Ann H. Klopp

In Fig 4, the panels A and B are identical. Please see the correct Fig 4 here.

**Fig 4:**
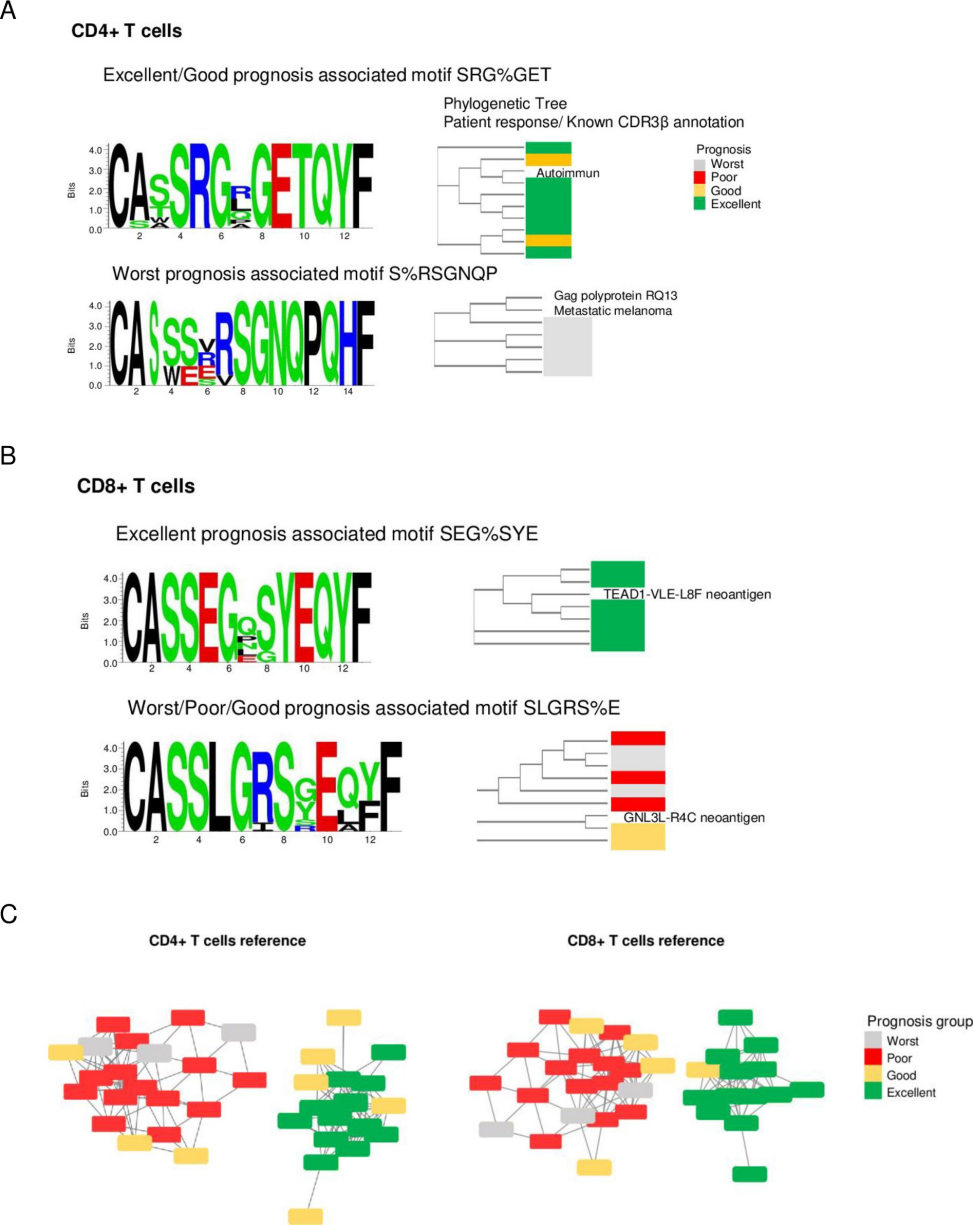
Ascitic fluid cell CDR3β motifs associated with prognosis. (A) Examples of CD4+ T cell receptor (TCR) motifs associated with different prognoses. (B) Examples of CD8+ TCR motifs associated with different prognoses. The figures show the logo and phylogenetic tree of the clustered CDR3β peptides for each motif. The phylogenetic tree also includes peptides found as the best hit from McPAS; the complete set of motifs associated with excellent or poor/worst prognosis is shown in S7A Table. (C) Association networks constructed to form the cluster of motifs associated with response to platinum therapy. Nodes in the networks indicate individual patients; the color of the node indicates prognosis. Edges in the networks indicate an association between a pair of samples if they share GLIPH specificity groups associated with prognosis. The networks validated the statistical algorithm used to identify prognosis-associated specificity groups.
