# Supplementary figures and images for: Immune environment and antigen specificity of the T cell receptor repertoire of malignant ascites in ovarian cancer
Source: PLoS One. 2023 Jan 6;18(1):e0279590. doi: 10.1371/journal.pone.0279590 (PMC9821423; doi:10.1371/journal.pone.0279590)

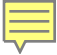

A

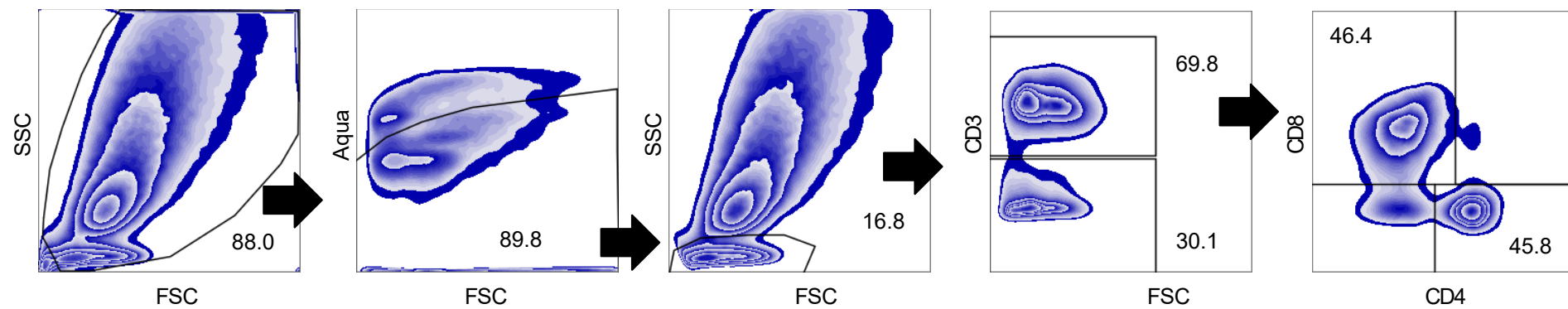

B

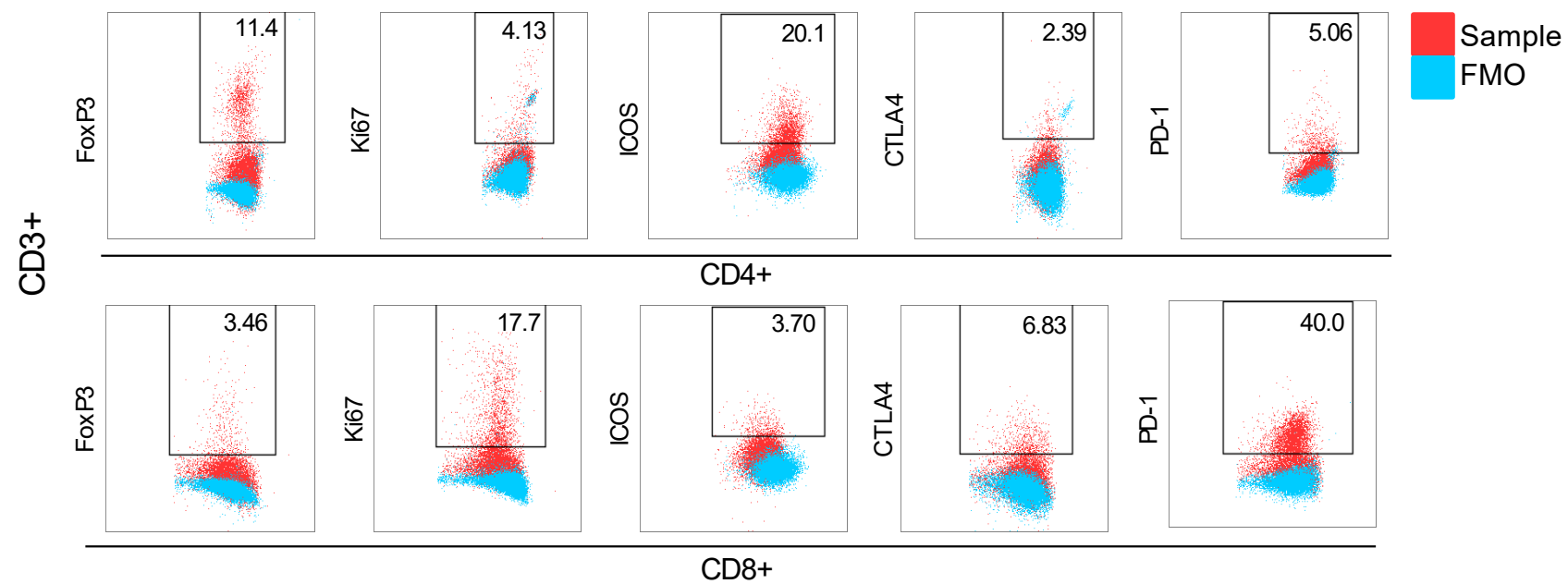

Supplement: S1 Fig — Cells collected from ascites were stained with a 9-color flow cytometry panel and analyzed in an X-20 Fortessa flow cytometer. (A) The gating strategy is depicted starting with the detection of lymphocytes, followed by live cells and CD3+ T cells that are separated according to CD4 and CD8 expression. (B) Fluorescence-minus-one (FMO) staining is shown for the different markers analyzed on CD4+ and CD8+ T cells. (PDF) [file pone.0279590.s001.pdf]

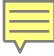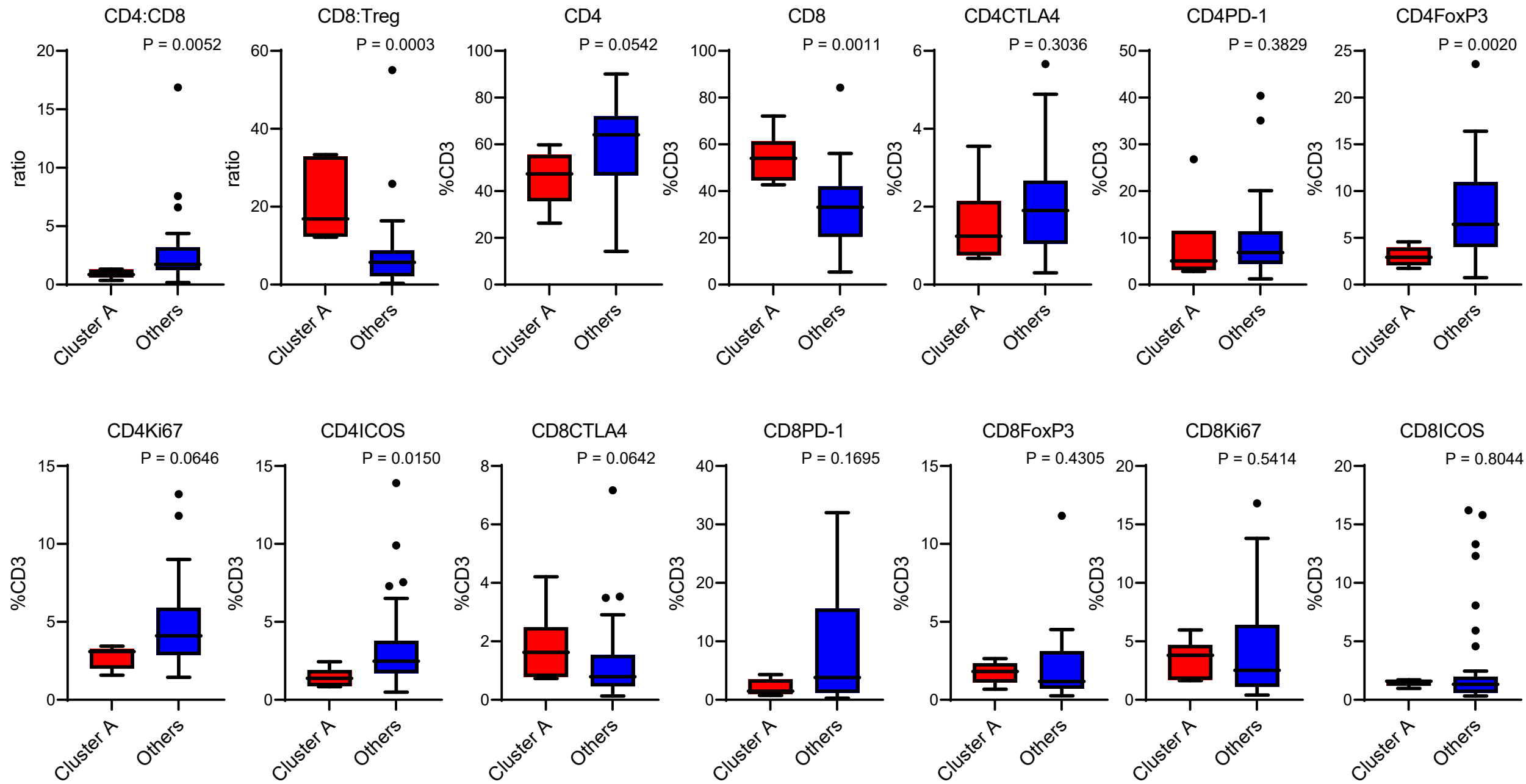

Supplement: S2 Fig — (PDF) [file pone.0279590.s002.pdf]

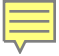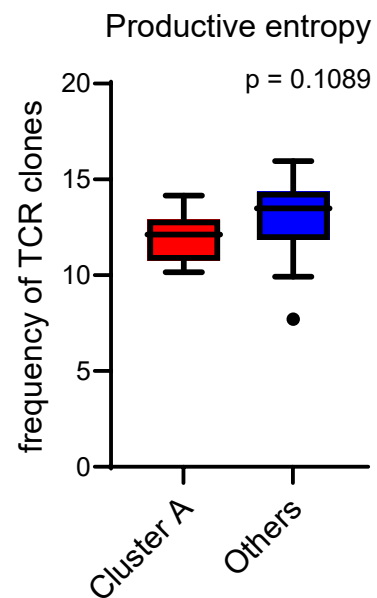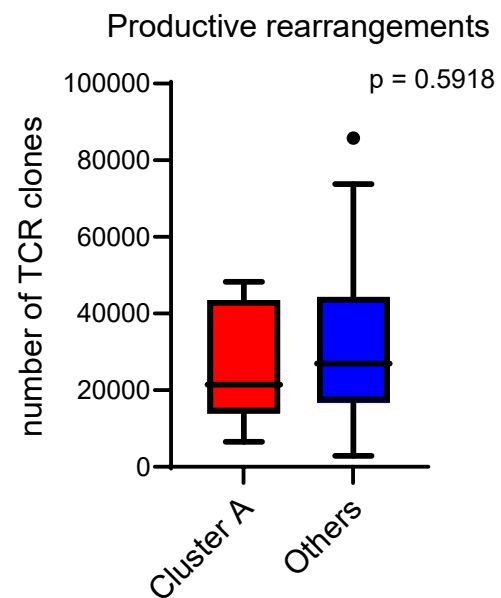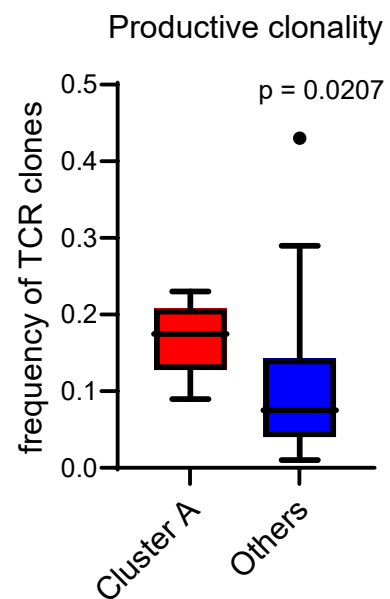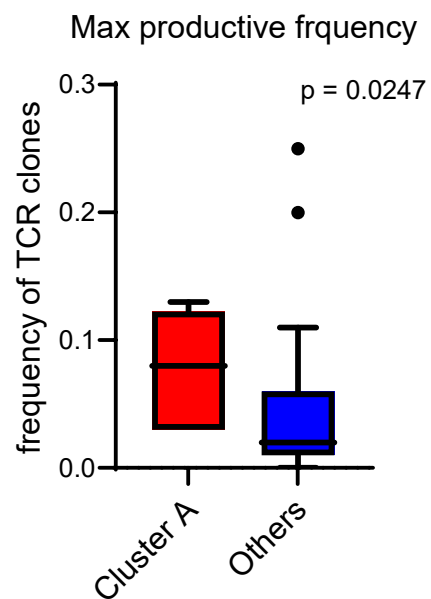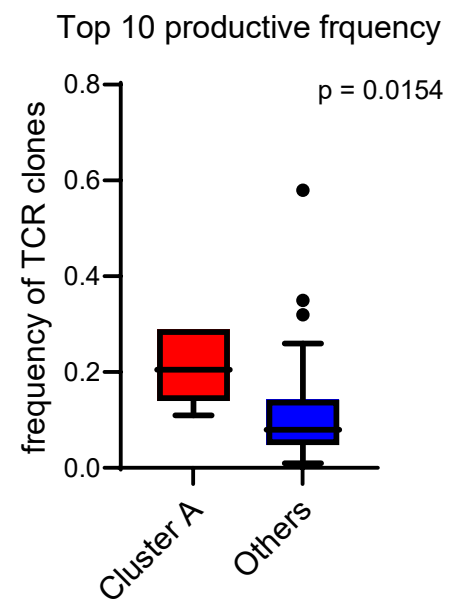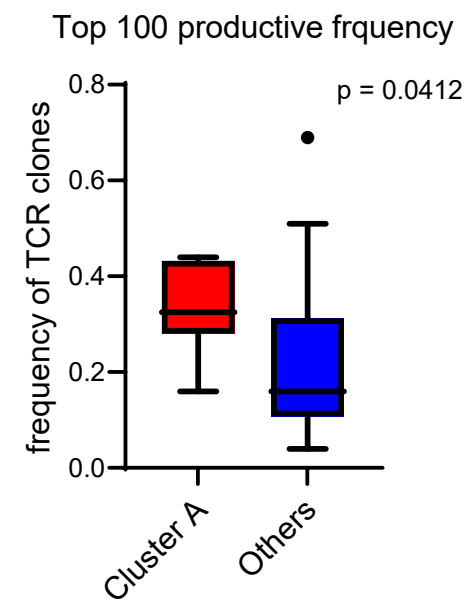

Supplement: S3 Fig — (PDF) [file pone.0279590.s003.pdf]

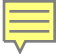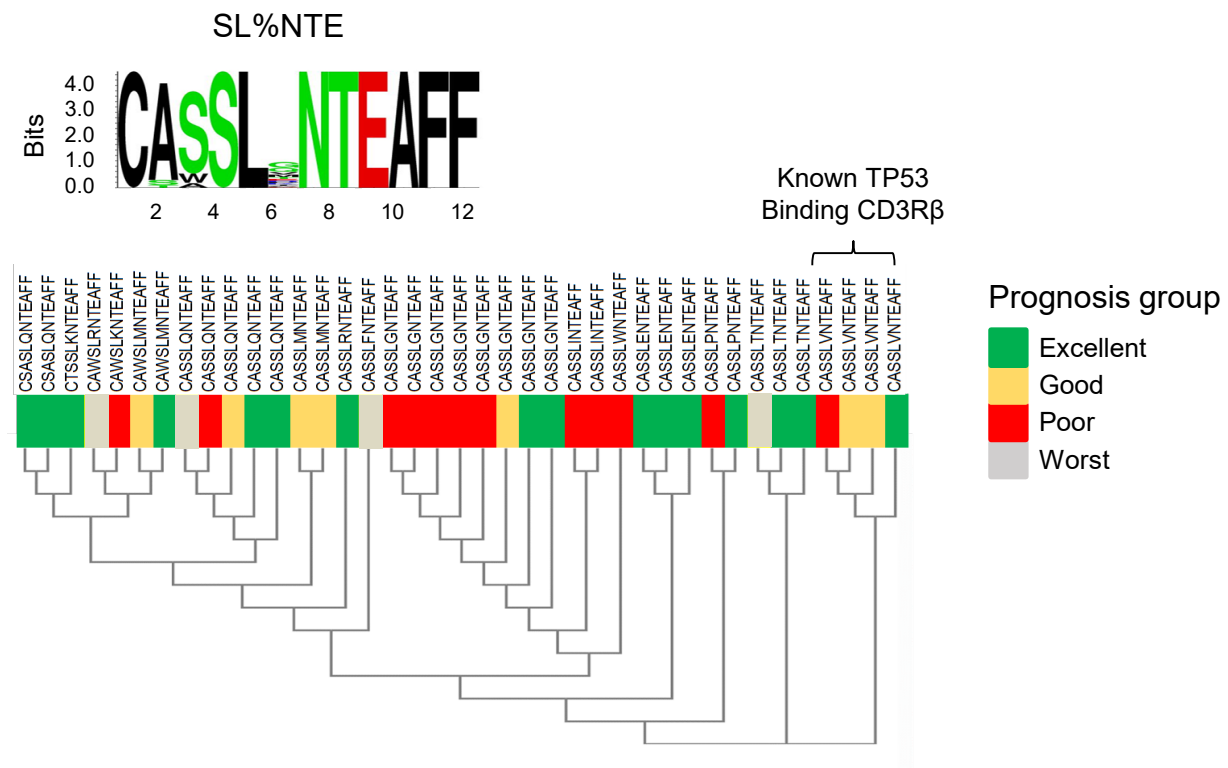

Supplement: S5 Fig — (PDF) [file pone.0279590.s005.pdf]

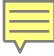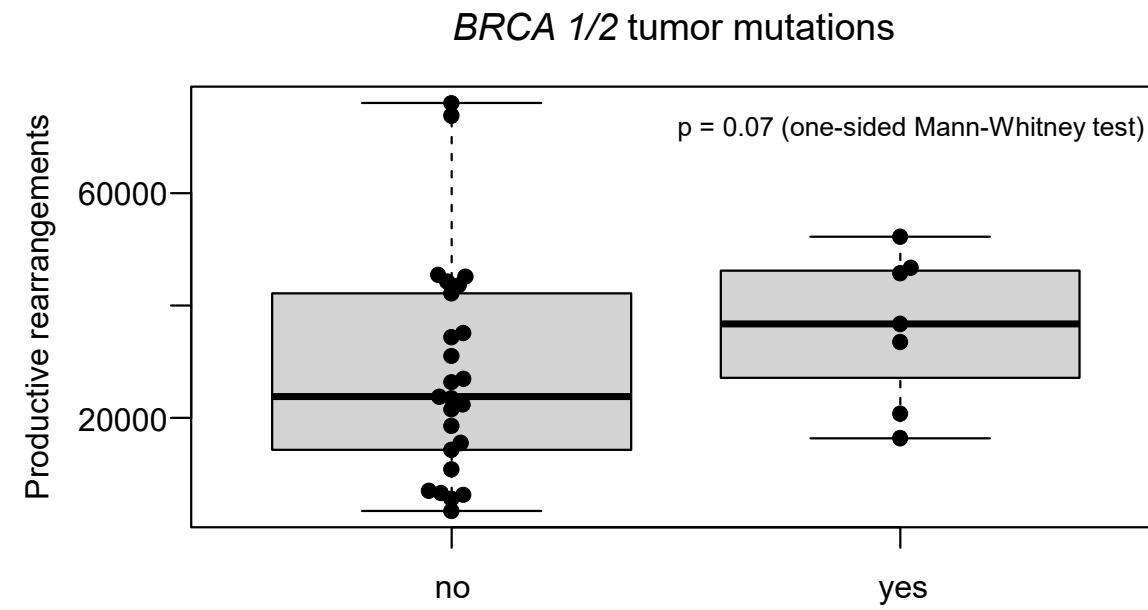

Supplement: S6 Fig — (PDF) [file pone.0279590.s006.pdf]

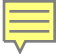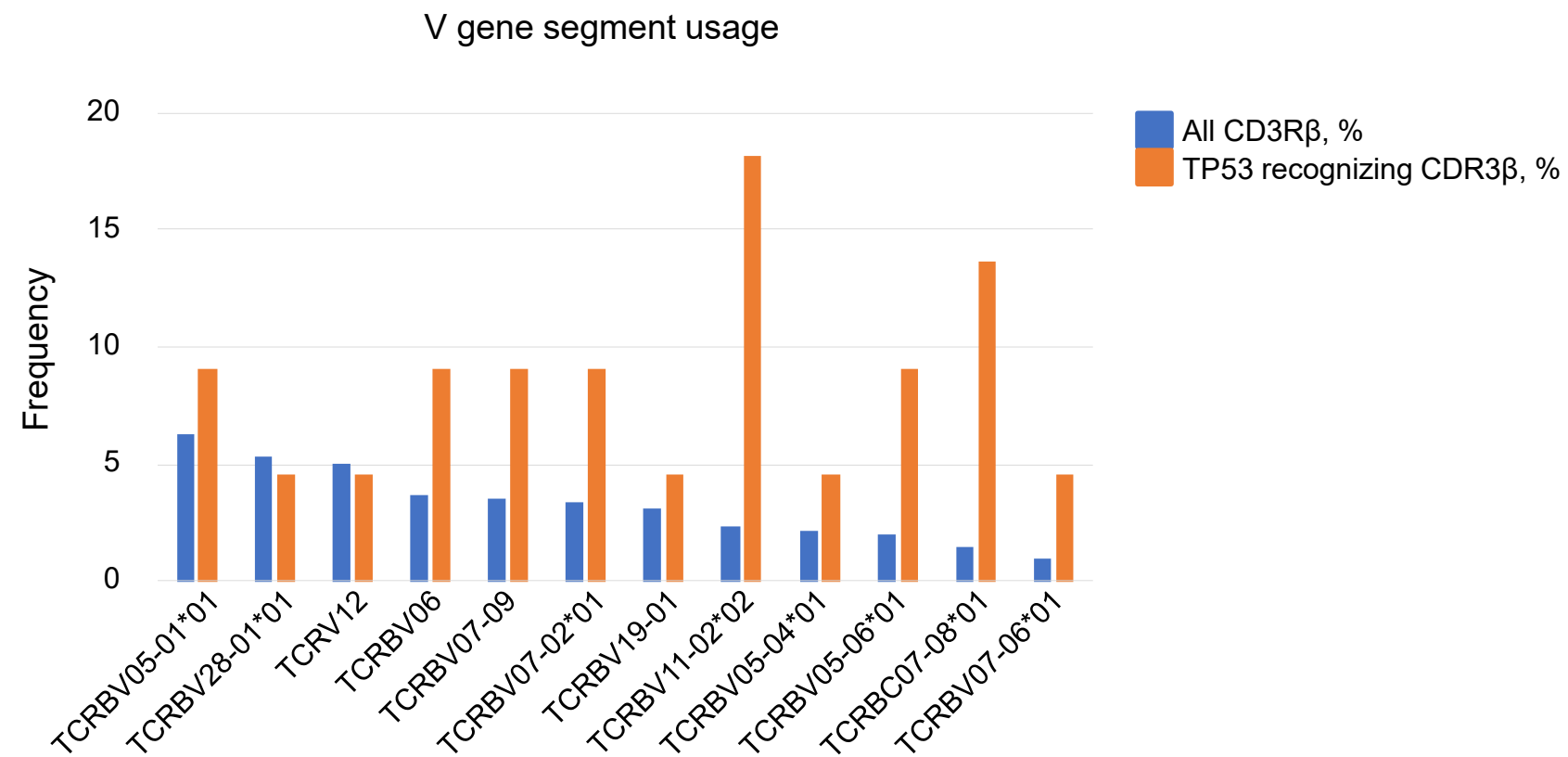

Supplement: S7 Fig — (PDF) [file pone.0279590.s007.pdf]
