# Supplementary material for: Immune environment and antigen specificity of the T cell receptor repertoire of malignant ascites in ovarian cancer
Source: PLoS One. 2023 Jan 6;18(1):e0279590. doi: 10.1371/journal.pone.0279590 (PMC9821423; doi:10.1371/journal.pone.0279590)
Supplement: S4 Fig — (PDF) [file pone.0279590.s004.pdf]

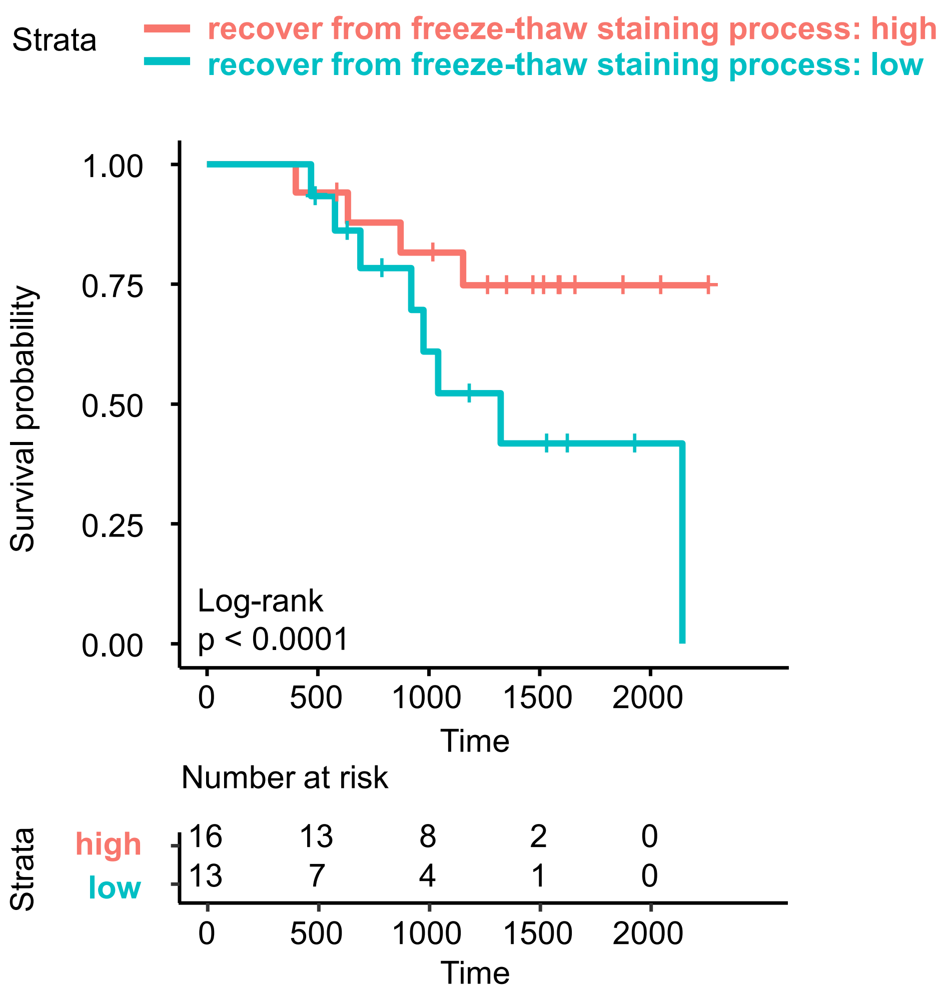

Strata
recover from freeze-thaw staining process: high
recover from freeze-thaw staining process: low
Survival probability
1.00
0.75
0.50
0.25
0.00
Log-rank
p < 0.0001
0
500
1000
1500
2000
Time
Number at risk
Strata
high
low
16 13 8 2 0
13 7 4 1 0
Time
