# Supplementary material for: Immune environment and antigen specificity of the T cell receptor repertoire of malignant ascites in ovarian cancer
Source: PLoS One. 2023 Jan 6;18(1):e0279590. doi: 10.1371/journal.pone.0279590 (PMC9821423; doi:10.1371/journal.pone.0279590)
Supplement: S1 Table — X indicates that data were obtained, and blank indicates missing data. Abbreviations: TCR, T cell receptor; FLOW, flow cytometry; HRD, homologous recombination deficiency; IHC, immunohistochemistry; ND, no data; SNV, single-nucleotide variant. (PDF) [file pone.0279590.s008.pdf]

Supplementary Table S1. TCR sequencing and T cell immune activity according to flow cytometry data for each patient, including p53, *TP53* mutation, *BRCA* mutation, and HRD status. X indicates that data were obtained, and blank indicates missing data.

Study no.	TCR data	FLOW data	Tumor tissue cell IHC: p53 (wild-type)	HRD status	*BRCA* mutation	*TP53* gene mutation	Standardized nomenclature (HGVS)	Location	DNA change	Protein change	dbSNP ID	COSMIC ID	Origin
2	X		ND	ND	Negative	ND	ND	ND	ND	ND	ND	ND	ND
3	X	X	ND	ND	Negative	ND	ND	ND	ND	ND	ND	ND	ND
6	X	X	ND	ND	ND	ND	ND	ND	ND	ND	ND	ND	ND
7	X	X	ND	ND	Negative	ND	ND	ND	ND	ND	ND	ND	ND
9	X		ND	ND	Negative	ND	ND	ND	ND	ND	ND	ND	ND
11	X	X	ND	ND	ND	ND	ND	ND	ND	ND	ND	ND	ND
12	X	X	Positive	ND	ND	ND	ND	ND	ND	ND	ND	ND	ND
13	X		Positive	ND	Negative	ND	ND	ND	ND	ND	ND	ND	ND
14	X	X	ND	ND	BRCA2	ND	ND	ND	ND	ND	ND	ND	ND
15	X	X	ND	ND	ND	Yes	NM_000546.5(*TP53*):c.637C>T p.R213*	Exon 6	SNV	Nonsense	ND	COSM10654	
16	X	X	ND	ND	Negative	ND	ND	ND	ND	ND	ND	ND	ND
17	X	X	ND	Negative	Negative	Yes	NM_000546.5(TP53):c.714dupT p.N239*	Exon 7	Duplication	Nonsense	ND	ND	Somatic
18	X	X	ND	ND	BRCA1	ND	ND	ND	ND	ND	ND	ND	ND
19	X	X	ND	ND	ND	ND	ND	ND	ND	ND	ND	ND	ND
20	X	X	ND	Negative	Negative	ND	ND	ND	ND	ND	ND	ND	ND
21	X	X	Positive	ND	ND	ND	ND	ND	ND	ND	ND	ND	ND
22	X	X	ND	ND	Negative	ND	ND	ND	ND	ND	ND	ND	ND
23	X	X	Positive	ND	Negative	ND	ND	ND	ND	ND	ND	ND	ND
24	X	X	ND	ND	ND	ND	ND	ND	ND	ND	ND	ND	ND
25	X	X	ND	ND	Negative	ND	ND	ND	ND	ND	ND	ND	ND
27	X	X	Positive	ND	Negative	Negative	Negative	Negative	Negative	Negative	Negative	Negative	Negative
28	X	X	Negative	ND	BRCA2	Yes	NM_000546.5(TP53):c.1024C>T p.R342*	Exon 10	SNV	Nonsense	ND	COSM11073	Somatic
29	X	X	Positive	Positive	Negative	ND	ND	ND	ND	ND	ND	ND	ND
30	X	X	ND	ND	Negative	Yes	NM_000546.5(TP53):c.476C>A p.A159D	Exon 5	SNV	Missense	ND	COSM11496	Somatic

31	X	X	Negative	ND	Negative	Yes	NM_000546.5(TP53):c.298C>T p.Q100*	Exon 4	SNV	Nonsense	ND	COSM44032	ND
32	X		Positive	ND	ND	ND	ND	ND	ND	ND	ND	ND	ND
34	X	X	ND	ND	Negative	Negative	Negative	Negative	Negative	Negative	Negative	Negative	Negative
35	X	X	Positive	ND	Negative	Negative	Negative	Negative	Negative	Negative	Negative	Negative	Negative
36	X	X	ND	ND	Negative	Yes	NM_000546.5(TP53):c.774A>T p.E258D	Exon 7	SNV	Missense	ND	COSM44962	Somatic
38	X		ND	ND	ND	ND	ND	ND	ND	ND	ND	ND	ND
39	X	X	Negative	ND	Negative	ND	ND	ND	ND	ND	ND	ND	ND
40	X	X	Negative	ND	Negative	ND	ND	ND	ND	ND	ND	ND	ND
42	X		ND	ND	ND	ND	ND	ND	ND	ND	ND	ND	ND
43	X	X	ND	ND	Negative	yes	NM_000546.5(TP53):c.646G>A p.V216M	Exon 6	SNV	Missense	ND	COSM10667	ND
44	X	X	ND	ND	ND	ND	ND	ND	ND	ND	ND	ND	ND
46	X	X	Positive	ND	BRCA1	ND	ND	ND	ND	ND	ND	ND	ND
47	X	X	Positive	ND	Negative	Negative	Negative	Negative	Negative	Negative	Negative	Negative	Negative
48	X	X	ND	ND	BRCA1	ND	ND	ND	ND	ND	ND	ND	ND
49	X		ND	ND	ND	ND	ND	ND	ND	ND	ND	ND	ND
50	X	X	Negative	ND	BRCA2	ND	ND	ND	ND	ND	ND	ND	ND
51			Positive	ND	ND	ND	ND	ND	ND	ND	ND	ND	ND
52	X	X	ND	ND	Negative	Yes	NM_000546.5(TP53):c.156del p.Q52fs	Exon 4	Deletion	Frameshift	ND	ND	ND
53	X	X	ND	ND	Negative	ND	ND	ND	ND	ND	ND	ND	ND
54	X	X	Negative	ND	BRCA1	Yes	NM_000546.5(TP53):c.637C>T p.R213*	Exon 6	SNV	Nonsense	Negative	COSM10654	Negative
55	X	X	Positive	ND	Negative	Negative	Negative	Negative	Negative	Negative	Negative	Negative	Negative
56			Negative	ND	ND	Yes	NM_000546.5(TP53):c.559+2T>A	Splice	Splice?	Unknown	Negative	Negative	Negative
57			Positive	ND	ND	ND	ND	ND	ND	ND	ND	ND	ND

Abbreviations: TCR, T cell receptor; FLOW, flow cytometry; HRD, homologous recombination deficiency; IHC, immunohistochemistry; ND, no data; SNV, single-nucleotide variant.
