# Supplementary material for: Immune environment and antigen specificity of the T cell receptor repertoire of malignant ascites in ovarian cancer
Source: PLoS One. 2023 Jan 6;18(1):e0279590. doi: 10.1371/journal.pone.0279590 (PMC9821423; doi:10.1371/journal.pone.0279590)
Supplement: S2 Table — (PDF) [file pone.0279590.s009.pdf]

Supplementary Table S2. Association of CD8+ T cell subsets and T cell receptor (TCR) characteristics with different prognoses using the Kruskal-Wallis rank-sum test and Dunn test.

Variable	Comparison	Kruskal-Wallis p	Adjusted p	p	Z
CD8+ T cell subset					
CD8+	Worst - Poor	0.105541437	0.321158488	0.267632074	0.61999034
CD8+	Worst - Good	0.105541437	0.269206059	0.179470706	0.91738408
CD8+	Poor - Good	0.105541437	0.288978034	0.288978034	0.556372742
CD8+	Worst - Excellent	0.105541437	0.115051835	0.038350612	1.770155601
CD8+	Poor - Excellent	0.105541437	0.100652581	0.01677543	2.125428669
CD8+	Good - Excellent	0.105541437	0.113812798	0.056906399	1.581285392
CD8+CTLA-4+	Worst - Poor	0.097765479	0.497988992	0.497988992	0.005040871
CD8+CTLA-4+	Worst - Good	0.097765479	0.349615981	0.291346651	0.549454908
CD8+CTLA-4+	Poor - Good	0.097765479	0.231328376	0.154218917	1.018505403
CD8+CTLA-4+	Worst - Excellent	0.097765479	0.187002089	0.093501045	1.319499763
CD8+CTLA-4+	Poor - Excellent	0.097765479	0.046471872	0.007745312	2.420700738
CD8+CTLA-4+	Good - Excellent	0.097765479	0.231416751	0.077138917	1.424582804
CD8+FoxP3+	Worst - Poor	0.035323886	0.085895461	0.05726364	1.578166863
CD8+FoxP3+	Worst - Good	0.035323886	0.044089762	0.014696587	2.178172795
CD8+FoxP3+	Poor - Good	0.035323886	0.156987825	0.130823187	1.122508314
CD8+FoxP3+	Worst - Excellent	0.035323886	0.02785566	0.00464261	2.601370179
CD8+FoxP3+	Poor - Excellent	0.035323886	0.057044519	0.02852226	1.902969545
CD8+FoxP3+	Good - Excellent	0.035323886	0.21037096	0.21037096	0.805134757
CD8+ICOS+	Worst - Poor	0.079204869	0.459833458	0.459833458	-0.1008533
CD8+ICOS+	Worst - Good	0.079204869	0.405330886	0.337775739	0.41854119
CD8+ICOS+	Poor - Good	0.079204869	0.248400567	0.165600378	0.971698113
CD8+ICOS+	Worst - Excellent	0.079204869	0.202836818	0.101418409	1.273510869
CD8+ICOS+	Poor - Excellent	0.079204869	0.034241667	0.005706944	2.529765172
CD8+ICOS+	Good - Excellent	0.079204869	0.17135768	0.057119227	1.579425669
CD8+Ki67+	Worst - Poor	0.061883971	0.360208394	0.360208394	0.357901821
CD8+Ki67+	Worst - Good	0.061883971	0.175255599	0.146046332	1.05354198
CD8+Ki67+	Poor - Good	0.061883971	0.19311351	0.096556755	1.30142357
CD8+Ki67+	Worst - Excellent	0.061883971	0.132376887	0.044125629	1.704695346

CD8+Ki67+	Poor - Excellent	0.061883971	0.038928202	0.006488034	2.484425455
CD8+Ki67+	Good - Excellent	0.061883971	0.169246826	0.112831217	1.211608094
CD8+PD-1+	Worst - Poor	0.340875511	0.254491724	0.127245862	-1.13950706
CD8+PD-1+	Worst - Good	0.340875511	0.263199274	0.175466183	-0.93278233
CD8+PD-1+	Poor - Good	0.340875511	0.419366305	0.349471921	0.386746562
CD8+PD-1+	Worst - Excellent	0.340875511	0.389182343	0.389182343	-0.28145077
CD8+PD-1+	Poor - Excellent	0.340875511	0.351583925	0.058597321	1.566658048
CD8+PD-1+	Good - Excellent	0.340875511	0.352006432	0.117335477	1.188412448
CD8/Treg	Worst - Poor	0.048682142	0.365905214	0.365905214	0.342718254
CD8/Treg	Worst - Good	0.048682142	0.182326221	0.151938517	1.028154762
CD8/Treg	Poor - Good	0.048682142	0.149794054	0.099862703	1.282334287
CD8/Treg	Worst - Excellent	0.048682142	0.120671479	0.040223826	1.748094573
CD8/Treg	Poor - Excellent	0.048682142	0.02861522	0.004769203	2.592128748
CD8/Treg	Good - Excellent	0.048682142	0.180902606	0.090451303	1.337981074
TCR characteristic					
Productive rearrangements	Worst - Poor	0.651865552	0.365502798	0.365502798	-0.34378817
Productive rearrangements	Worst - Good	0.651865552	0.467751624	0.233875812	-0.72614216
Productive rearrangements	Poor - Good	0.651865552	0.391785378	0.261190252	-0.63968024
Productive rearrangements	Worst - Excellent	0.651865552	0.502301997	0.167433999	-0.96435495
Productive rearrangements	Poor - Excellent	0.651865552	0.88866588	0.14811098	-1.04456955
Productive rearrangements	Good - Excellent	0.651865552	0.419981129	0.349984275	-0.38536292
Productive clonality	Worst - Poor	0.265943669	0.488358924	0.488358924	0.029183992
Productive clonality	Worst - Good	0.265943669	0.301262949	0.251052458	0.671181495
Productive clonality	Poor - Good	0.265943669	0.285546898	0.142773449	1.067941507
Productive clonality	Worst - Excellent	0.265943669	0.380746148	0.126915383	1.141094102
Productive clonality	Poor - Excellent	0.265943669	0.183776085	0.030629348	1.871623607
Productive clonality	Good - Excellent	0.265943669	0.331555619	0.221037079	0.768695397
Maximum productive frequency	Worst - Poor	0.10360702	0.38322648	0.3193554	-0.46950208
Maximum productive frequency	Worst - Good	0.10360702	0.393556316	0.393556316	0.270061901
Maximum productive frequency	Poor - Good	0.10360702	0.331078486	0.110359495	1.224618509
Maximum productive frequency	Worst - Excellent	0.10360702	0.239224639	0.159483092	0.996584598
Maximum productive frequency	Poor - Excellent	0.10360702	0.040784832	0.006797472	2.467791603
Maximum productive frequency	Good - Excellent	0.10360702	0.231290153	0.115645077	1.197042081

Productive entropy	Worst - Poor	0.204460985	0.486055217	0.486055217	0.034961509
Productive entropy	Worst - Good	0.204460985	0.318625867	0.265521556	-0.62641448
Productive entropy	Poor - Good	0.204460985	0.271571652	0.135785826	-1.09945041
Productive entropy	Worst - Excellent	0.204460985	0.357029601	0.119009867	-1.17995093
Productive entropy	Poor - Excellent	0.204460985	0.122563878	0.020427313	-2.04500236
Productive entropy	Good - Excellent	0.204460985	0.273176524	0.182117683	-0.90732423
Top 10 productive frequency	Worst - Poor	0.124073732	0.381764244	0.381764244	-0.30085051
Top 10 productive frequency	Worst - Good	0.124073732	0.356538631	0.297115526	0.532714754
Top 10 productive frequency	Poor - Good	0.124073732	0.250078626	0.083359542	1.382823198
Top 10 productive frequency	Worst - Excellent	0.124073732	0.276583039	0.138291519	1.088027339
Top 10 productive frequency	Poor - Excellent	0.124073732	0.058188239	0.00969804	2.337829787
Top 10 productive frequency	Good - Excellent	0.124073732	0.271601027	0.181067351	0.911304982
Top 100 productive frequency	Worst - Poor	0.209454178	0.481959923	0.481959923	-0.04523519
Top 100 productive frequency	Worst - Good	0.209454178	0.33537277	0.223581847	0.760152065
Top 100 productive frequency	Poor - Good	0.209454178	0.270943712	0.090314571	1.33882042
Top 100 productive frequency	Worst - Excellent	0.209454178	0.25749198	0.12874599	1.132338904
Top 100 productive frequency	Poor - Excellent	0.209454178	0.142386505	0.023731084	1.982152561
Top 100 productive frequency	Good - Excellent	0.209454178	0.326597584	0.272164653	0.606279293
