# Supplementary material for: Immune environment and antigen specificity of the T cell receptor repertoire of malignant ascites in ovarian cancer
Source: PLoS One. 2023 Jan 6;18(1):e0279590. doi: 10.1371/journal.pone.0279590 (PMC9821423; doi:10.1371/journal.pone.0279590)
Supplement: S3 Table — (PDF) [file pone.0279590.s010.pdf]

Supplementary Table S3. Pairwise association between T cell receptor (TCR) characteristics and T cell subsets identified by flow cytometry, using the Pearson correlation test (no adjustment).

TCR characteristic	T cell subset	p	R (Pearson)	Adjusted p
Productive entropy	CD8/Treg	1.71E-05	-0.643943744	0.003277673
Top 100 productive frequency	CD8/Treg	0.000171939	0.579323654	0.032840423
Top 10 productive frequency	CD8/Treg	0.000983889	0.519510691	0.184971156
Productive clonality	CD8/Treg	0.000990748	0.519248793	0.185269823
Productive rearrangements	CD4+FoxP3+	0.004896721	0.45275443	0.886306443
Top 10 productive frequency	CD8+CTLA4+	0.005896321	0.444098286	1
Productive rearrangements	CD8/Treg	0.006224375	-0.441533321	1
Top 100 productive frequency	CD8+CTLA4+	0.008686301	0.425306412	1
Productive clonality	CD8+CTLA4+	0.010212151	0.417140495	1
Productive clonality	CD8+	0.010720958	0.414648538	1
Productive rearrangements	CD4+	0.01353575	0.402441952	1
Top 10 productive frequency	CD8+	0.015527981	0.395044021	1
Top 100 productive frequency	CD8+	0.021819647	0.375998554	1
Productive clonality	CD8+Ki67+	0.028771806	0.359692582	1
Top 100 productive frequency	CD8+Ki67+	0.038436679	0.341740542	1
Top 10 productive frequency	CD8+Ki67+	0.046736241	0.329062798	1
Productive rearrangements	CD4+Ki67+	0.057147755	0.315505334	1
Productive entropy	CD8+CTLA4+	0.062312901	-0.309500109	1
Productive entropy	CD4+FoxP3+	0.067661124	0.303683617	1
Top 10 productive frequency	CD8+ICOS+	0.072908952	0.298317963	1
Productive rearrangements	CD3+	0.088783758	0.28373793	1
Productive clonality	CD3+	0.089897235	0.282793491	1
Productive clonality	CD8+ICOS+	0.11302035	0.264946638	1
Top 10 productive frequency	CD3+	0.12064194	0.259674416	1
Top 100 productive frequency	CD8+ICOS+	0.12959135	0.253792779	1
Productive rearrangements	CD4+PD-1+	0.143332962	0.24532242	1
Top 10 productive frequency	CD4+CTLA4+	0.153507706	0.239427674	1
Top 100 productive frequency	CD8+PD-1+	0.158181081	0.236815235	1
Top 10 productive frequency	CD8+PD-1+	0.159700505	0.235977932	1

Productive clonality	CD8+PD-1+	0.162953962	0.234204243	1
Productive clonality	CD4+Ki67+	0.164940197	0.233133933	1
Top 10 productive frequency	CD4+Ki67+	0.176010693	0.227332178	1
Productive entropy	CD8+	0.182272103	-0.224165383	1
Productive clonality	CD8+FoxP3+	0.194958085	0.217978101	1
Productive clonality	CD4+PD-1+	0.195838456	0.217559363	1
Top 10 productive frequency	CD8+FoxP3+	0.200066558	0.215566473	1
Top 100 productive frequency	CD3+	0.209136302	0.211388555	1
Top 100 productive frequency	CD8+FoxP3+	0.21090605	0.210588071	1
Productive clonality	CD4+CTLA4+	0.238537451	0.198648581	1
Productive entropy	CD8+ICOS+	0.255384005	-0.191823378	1
Productive rearrangements	CD4+ICOS+	0.277224221	0.183406355	1
Top 10 productive frequency	CD4+ICOS+	0.310069208	0.171523882	1
Top 10 productive frequency	CD4+PD-1+	0.313225592	0.170425093	1
Productive clonality	CD4+	0.31527752	0.169714518	1
Top 100 productive frequency	CD4+Ki67+	0.319042797	0.168418134	1
Top 100 productive frequency	CD4+PD-1+	0.330316384	0.164592851	1
Productive clonality	CD4+ICOS+	0.335784035	0.162766736	1
Top 100 productive frequency	CD4+CTLA4+	0.33905503	0.161683017	1
Productive entropy	CD8+Ki67+	0.365806259	-0.153049813	1
Top 10 productive frequency	CD4+	0.407185437	0.140405388	1
Productive rearrangements	CD4+CTLA4+	0.407926573	0.140185867	1
Top 100 productive frequency	CD4+ICOS+	0.454107483	0.12692392	1
Top 100 productive frequency	CD4+FoxP3+	0.529301551	-0.106787727	1
Productive entropy	CD8+PD-1+	0.540476708	-0.103921127	1
Productive rearrangements	CD8+	0.546204152	0.102462969	1
Productive entropy	CD8+FoxP3+	0.581434329	-0.093646266	1
Productive entropy	CD4+	0.584584003	0.09287004	1
Productive rearrangements	CD8+CTLA4+	0.589997003	-0.091540353	1
Productive rearrangements	CD8+Ki67+	0.626856335	0.082622534	1
Top 100 productive frequency	CD4+	0.635357382	0.080597243	1
Productive entropy	CD4+CTLA4+	0.794347573	-0.044355061	1
Top 10 productive frequency	CD4+FoxP3+	0.812227759	-0.040425027	1

Productive entropy	CD4+Ki67+	0.853050368	0.031525763	1
Productive entropy	CD3+	0.872851984	-0.027239999	1
Productive clonality	CD4+FoxP3+	0.899208846	-0.021560528	1
Productive entropy	CD4+PD-1+	0.901810628	0.021001229	1
Productive rearrangements	CD8+ICOS+	0.923984534	-0.016242745	1
Productive entropy	CD4+ICOS+	0.950883133	-0.010486266	1
Productive rearrangements	CD8+FoxP3+	0.981733645	0.003897766	1
Productive rearrangements	CD8+PD-1+	0.995842969	-0.000886978	1
