# Supplementary material for: Immune environment and antigen specificity of the T cell receptor repertoire of malignant ascites in ovarian cancer
Source: PLoS One. 2023 Jan 6;18(1):e0279590. doi: 10.1371/journal.pone.0279590 (PMC9821423; doi:10.1371/journal.pone.0279590)
Supplement: S4 Table — Significance is labeled as *<0.05, **<0.01, ***<0.001. (PDF) [file pone.0279590.s011.pdf]

Supplementary Table S4. Cox proportional hazards models of flow cytometry and T cell receptor characteristics with a prognostic value independent of debulking status.

Characteristic	No. of covariates	Covariate name	Coef.	Exp (coef.)	SE (coef.)	z	Pr (>|z|)	Significance label	Hazard ratio	Concordance	Likelihood ratio test p value
CD8/Treg	1	CD8/Treg	0.5226	1.6864	0.1886	2.771	0.00559	**	1.686		0.01
Debulking status	1	Debulking status	1.7617	5.8223	0.4646	3.792	0.00015	***	5.822	0.679 (SE=0.053)	6e-04
CD8/Treg	2	CD8/Treg	0.6907	1.9952	0.2086	3.311	0.000929	***	1.995	0.737 (SE=0.081)	7e-04
		Debulking status	2.0317	7.6273	0.6398	3.176	0.001495	**	7.627		
Productive clonality	2	Productive clonality	0.654	1.9231	0.2215	2.952	0.00316	**	1.923	0.747 (SE=0.066)	4e-04
		Debulking status	1.6913	5.4265	0.5255	3.219	0.00129	**	5.427		
Maximum productive frequency	2	Maximum productive frequency	0.5862	1.7971	0.2095	2.798	0.00514	**	1.797	0.776 (SE=0.054)	5e-04
		Debulking status	1.2373	3.4463	0.5637	2.195	0.02816	*	3.446		
Productive entropy	2	Productive entropy	-0.6256	0.5349	0.2465	-2.538	0.011146	*	0.5349	0.74 (SE=0.064)	8e-04
		Debulking status	1.8073	6.0938	0.5334	3.388	0.000704	***	6.0938		
Top 10 productive frequency	2	Top 10 productive frequency	0.6577	1.9304	0.2034	3.234	0.00122	**	1.93	0.762 (SE=0.06)	2e-04
		Debulking status	1.7146	5.5542	0.5276	3.25	0.00116	**	5.554		
Top 100 productive frequency	2	Top 100 productive frequency	0.6821	1.9781	0.2481	2.749	0.005975	**	1.978	0.749 (SE=0.064)	5e-04
		Debulking status	1.8353	6.2668	0.5368	3.419	0.000629	***	6.267		

Significance is labeled as *<0.05, **<0.01, ***<0.001.
