# Supplementary material for: Immune environment and antigen specificity of the T cell receptor repertoire of malignant ascites in ovarian cancer
Source: PLoS One. 2023 Jan 6;18(1):e0279590. doi: 10.1371/journal.pone.0279590 (PMC9821423; doi:10.1371/journal.pone.0279590)
Supplement: S5 Table — (PDF) [file pone.0279590.s012.pdf]

Supplementary Table S5. Association of pathologic conditions inferred from McPAS annotation with different prognoses according to the Kruskal-Wallis rank-sum test and Dunn test.

Pathologic condition	Comparison	Kruskal-Wallis p	Adjusted p	p	Z
Acute myeloid leukemia	Worst - Poor	0.425275991	0.357585357	0.178793	-0.91998
Acute myeloid leukemia	Worst - Good	0.425275991	0.418134856	0.069689	-1.47811
Acute myeloid leukemia	Poor - Good	0.425275991	0.305249602	0.2035	-0.82919
Acute myeloid leukemia	Worst - Excellent	0.425275991	0.225535268	0.075178	-1.43827
Acute myeloid leukemia	Poor - Excellent	0.425275991	0.26213797	0.218448	-0.77744
Acute myeloid leukemia	Good - Excellent	0.425275991	0.498172398	0.498172	0.004581
Allergy	Worst - Poor	0.556103413	0.322013257	0.322013	-0.46208
Allergy	Worst - Good	0.556103413	0.41479789	0.207399	-0.81548
Allergy	Poor - Good	0.556103413	0.414957266	0.276638	-0.59286
Allergy	Worst - Excellent	0.556103413	0.376061866	0.125354	-1.14863
Allergy	Poor - Excellent	0.556103413	0.74347967	0.123913	-1.15564
Allergy	Good - Excellent	0.556103413	0.353028412	0.29419	-0.54118
Alzheimer disease	Worst - Poor	0.90568003	0.817671457	0.272557	-0.6051
Alzheimer disease	Worst - Good	0.90568003	0.581931836	0.290966	-0.55057
Alzheimer disease	Poor - Good	0.90568003	0.46655847	0.466558	0.083924
Alzheimer disease	Worst - Excellent	0.90568003	1	0.229438	-0.7407
Alzheimer disease	Poor - Excellent	0.90568003	0.491671586	0.409726	-0.22825
Alzheimer disease	Good - Excellent	0.90568003	0.568615271	0.379077	-0.30791
Breast cancer	Worst - Poor	0.828800292	0.471254738	0.471255	0.072116
Breast cancer	Worst - Good	0.828800292	0.92053525	0.306845	-0.50481
Breast cancer	Poor - Good	0.828800292	1	0.18083	-0.91221
Breast cancer	Worst - Excellent	0.828800292	0.497045659	0.414205	-0.21674
Breast cancer	Poor - Excellent	0.828800292	0.482485129	0.321657	-0.46307
Breast cancer	Good - Excellent	0.828800292	0.640357239	0.320179	0.467199
Calcified aortic stenosis disease	Worst - Poor	0.197863144	0.136004226	0.022667	-2.00154
Calcified aortic stenosis disease	Worst - Good	0.197863144	0.183844901	0.153204	-1.02279
Calcified aortic stenosis disease	Poor - Good	0.197863144	0.15063689	0.075318	1.437285
Calcified aortic stenosis disease	Worst - Excellent	0.197863144	0.210437442	0.140292	-1.07901
Calcified aortic stenosis disease	Poor - Excellent	0.197863144	0.223612746	0.074538	1.442806

Calcified aortic stenosis disease	Good - Excellent	0.197863144	0.478379029	0.478379	-0.05422
Carcinoma	Worst - Poor	0.10698024	0.14303646	0.095358	-1.30847
Carcinoma	Worst - Good	0.10698024	0.079619945	0.01327	-2.21822
Carcinoma	Poor - Good	0.10698024	0.163317204	0.081659	-1.394
Carcinoma	Worst - Excellent	0.10698024	0.174615255	0.145513	-1.05587
Carcinoma	Poor - Excellent	0.10698024	0.333780475	0.33378	0.429498
Carcinoma	Good - Excellent	0.10698024	0.096322359	0.032107	1.850685
Celiac disease	Worst - Poor	0.477917853	0.423543903	0.352953	-0.37736
Celiac disease	Worst - Good	0.477917853	0.439066617	0.292711	-0.54548
Celiac disease	Poor - Good	0.477917853	0.388282956	0.388283	-0.2838
Celiac disease	Worst - Excellent	0.477917853	0.366555774	0.122185	-1.16413
Celiac disease	Poor - Excellent	0.477917853	0.55617485	0.092696	-1.32434
Celiac disease	Good - Excellent	0.477917853	0.309733013	0.154867	-1.01578
Cervical intraepithelial neoplasia	Worst - Poor	0.614934936	0.359396771	0.179698	-0.91652
Cervical intraepithelial neoplasia	Worst - Good	0.614934936	0.5	0.5	0
Cervical intraepithelial neoplasia	Poor - Good	0.614934936	0.539095156	0.179698	0.916515
Cervical intraepithelial neoplasia	Worst - Excellent	0.614934936	0.6	0.5	0
Cervical intraepithelial neoplasia	Poor - Excellent	0.614934936	1	0.179698	0.916515
Cervical intraepithelial neoplasia	Good - Excellent	0.614934936	0.75	0.5	0
Clear cell renal carcinoma	Worst - Poor	0.767466434	0.459849386	0.459849	-0.10081
Clear cell renal carcinoma	Worst - Good	0.767466434	0.414142043	0.345118	-0.39853
Clear cell renal carcinoma	Poor - Good	0.767466434	0.61973666	0.309868	-0.49622
Clear cell renal carcinoma	Worst - Excellent	0.767466434	0.741174661	0.247058	-0.68378
Clear cell renal carcinoma	Poor - Excellent	0.767466434	0.97937418	0.163229	-0.98127
Clear cell renal carcinoma	Good - Excellent	0.767466434	0.480532476	0.320355	-0.46671
Colorectal cancer	Worst - Poor	0.992840865	0.491282814	0.491283	0.021852
Colorectal cancer	Worst - Good	0.992840865	0.568688998	0.473907	-0.06545
Colorectal cancer	Poor - Good	0.992840865	0.884760006	0.44238	-0.14494
Colorectal cancer	Worst - Excellent	0.992840865	1	0.439212	-0.15297
Colorectal cancer	Poor - Excellent	0.992840865	1	0.384277	-0.29427
Colorectal cancer	Good - Excellent	0.992840865	0.664227732	0.442818	-0.14383
Cytomegalovirus	Worst - Poor	0.711727755	0.437474949	0.218737	-0.77646
Cytomegalovirus	Worst - Good	0.711727755	0.549141105	0.183047	-0.90381

Cytomegalovirus	Poor - Good	0.711727755	0.412759136	0.412759	-0.22045
Cytomegalovirus	Worst - Excellent	0.711727755	0.769336773	0.128223	-1.13483
Cytomegalovirus	Poor - Excellent	0.711727755	0.409770068	0.27318	-0.60322
Cytomegalovirus	Good - Excellent	0.711727755	0.426161746	0.355135	-0.37149
Diabetes type 1	Worst - Poor	0.703417928	0.428174398	0.28545	-0.56673
Diabetes type 1	Worst - Good	0.703417928	0.420417101	0.210209	-0.8057
Diabetes type 1	Poor - Good	0.703417928	0.411846865	0.343206	-0.40373
Diabetes type 1	Worst - Excellent	0.703417928	0.878577698	0.14643	-1.05187
Diabetes type 1	Poor - Excellent	0.703417928	0.621221824	0.207074	-0.81662
Diabetes type 1	Good - Excellent	0.703417928	0.345454763	0.345455	-0.39762
Diabetes type 2	Worst - Poor	0.225227537	0.298544722	0.049757	1.64721
Diabetes type 2	Worst - Good	0.225227537	0.228878688	0.190732	0.875201
Diabetes type 2	Poor - Good	0.225227537	0.156124492	0.052041	-1.62537
Diabetes type 2	Worst - Excellent	0.225227537	0.190492224	0.095246	1.309124
Diabetes type 2	Poor - Excellent	0.225227537	0.224367091	0.224367	-0.75753
Diabetes type 2	Good - Excellent	0.225227537	0.248929506	0.165953	0.970282
Epithelial ovarian cancer	Worst - Poor	0.02937921	0.236442655	0.157628	-1.00425
Epithelial ovarian cancer	Worst - Good	0.02937921	0.252998278	0.210832	-0.80354
Epithelial ovarian cancer	Poor - Good	0.02937921	0.366796237	0.366796	0.340351
Epithelial ovarian cancer	Worst - Excellent	0.02937921	0.030875382	0.010292	-2.31554
Epithelial ovarian cancer	Poor - Excellent	0.02937921	0.044041605	0.022021	-2.01369
Epithelial ovarian cancer	Good - Excellent	0.02937921	0.043091964	0.007182	-2.44803
Epstein-Barr virus	Worst - Poor	0.280544319	0.275644707	0.045941	-1.68555
Epstein-Barr virus	Worst - Good	0.280544319	0.225590103	0.150393	-1.03475
Epstein-Barr virus	Poor - Good	0.280544319	0.287585957	0.143793	1.063432
Epstein-Barr virus	Worst - Excellent	0.280544319	0.150931673	0.050311	-1.64185
Epstein-Barr virus	Poor - Excellent	0.280544319	0.470677696	0.470678	0.073566
Epstein-Barr virus	Good - Excellent	0.280544319	0.192940731	0.160784	-0.99124
Hepatitis C virus	Worst - Poor	0.205051547	0.127310544	0.042437	-1.72308
Hepatitis C virus	Worst - Good	0.205051547	0.203719148	0.10186	-1.27103
Hepatitis C virus	Poor - Good	0.205051547	0.345593952	0.287995	0.559252
Hepatitis C virus	Worst - Excellent	0.205051547	0.116714588	0.019452	-2.06519
Hepatitis C virus	Poor - Excellent	0.205051547	0.350347062	0.350347	-0.38438

Hepatitis C virus	Good - Excellent	0.205051547	0.249763076	0.166509	-0.96805
Human T cell leukemia virus type 1	Worst - Poor	0.715917835	0.477833482	0.398195	-0.25802
Human T cell leukemia virus type 1	Worst - Good	0.715917835	0.344871417	0.229914	-0.73913
Human T cell leukemia virus type 1	Poor - Good	0.715917835	0.633011746	0.211004	-0.80294
Human T cell leukemia virus type 1	Worst - Excellent	0.715917835	0.433735392	0.216868	-0.78282
Human T cell leukemia virus type 1	Poor - Excellent	0.715917835	1	0.188522	-0.88336
Human T cell leukemia virus type 1	Good - Excellent	0.715917835	0.474524123	0.474524	-0.0639
Human immunodeficiency virus	Worst - Poor	0.361597363	0.326666508	0.272222	-0.60611
Human immunodeficiency virus	Worst - Good	0.361597363	0.209621	0.104811	-1.25461
Human immunodeficiency virus	Poor - Good	0.361597363	0.208373354	0.138916	-1.0852
Human immunodeficiency virus	Worst - Excellent	0.361597363	0.51932658	0.086554	-1.36228
Human immunodeficiency virus	Poor - Excellent	0.361597363	0.304615618	0.101539	-1.27283
Human immunodeficiency virus	Good - Excellent	0.361597363	0.434930175	0.43493	-0.16384
IgG4-related disease	Worst - Poor	0.155898772	0.424978602	0.424979	-0.18917
IgG4-related disease	Worst - Good	0.155898772	0.334294605	0.278579	-0.58707
IgG4-related disease	Poor - Good	0.155898772	0.378169847	0.252113	-0.66785
IgG4-related disease	Worst - Excellent	0.155898772	0.160468836	0.080234	-1.4035
IgG4-related disease	Poor - Excellent	0.155898772	0.11498429	0.019164	-2.07133
IgG4-related disease	Good - Excellent	0.155898772	0.229743292	0.076581	-1.42845
Inflammatory bowel disease	Worst - Poor	0.939384489	0.691801071	0.345901	-0.39641
Inflammatory bowel disease	Worst - Good	0.939384489	0.583358936	0.388906	-0.28217
Inflammatory bowel disease	Poor - Good	0.939384489	0.426399672	0.4264	0.185548
Inflammatory bowel disease	Worst - Excellent	0.939384489	1	0.28836	-0.55818
Inflammatory bowel disease	Poor - Excellent	0.939384489	0.471234198	0.392695	-0.2723
Inflammatory bowel disease	Good - Excellent	0.939384489	0.976083434	0.325361	-0.45276
Influenza	Worst - Poor	0.552932942	0.470968122	0.470968	-0.07284
Influenza	Worst - Good	0.552932942	0.460307557	0.38359	-0.29607
Influenza	Poor - Good	0.552932942	0.532399395	0.354933	-0.37204
Influenza	Worst - Excellent	0.552932942	0.385278941	0.192639	-0.86821
Influenza	Poor - Excellent	0.552932942	0.541893216	0.090316	-1.33881
Influenza	Good - Excellent	0.552932942	0.51947985	0.17316	-0.94175
Lung cancer	Worst - Poor	0.825896326	0.496751506	0.41396	-0.21737
Lung cancer	Worst - Good	0.825896326	0.611601316	0.407734	0.233377

Lung cancer	Poor - Good	0.825896326	0.763294388	0.254431	0.660609
Lung cancer	Worst - Excellent	0.825896326	0.726562559	0.363281	-0.3497
Lung cancer	Poor - Excellent	0.825896326	0.42729044	0.42729	-0.18328
Lung cancer	Good - Excellent	0.825896326	1	0.182751	-0.90493
Mycobacterium tuberculosis	Worst - Poor	0.89623461	0.540179633	0.45015	0.125283
Mycobacterium tuberculosis	Worst - Good	0.89623461	0.450394783	0.450395	-0.12466
Mycobacterium tuberculosis	Poor - Good	0.89623461	1	0.339345	-0.41425
Mycobacterium tuberculosis	Worst - Excellent	0.89623461	0.556484983	0.37099	-0.32923
Mycobacterium tuberculosis	Poor - Excellent	0.89623461	1	0.222117	-0.76506
Mycobacterium tuberculosis	Good - Excellent	0.89623461	0.736486073	0.368243	-0.33651
Melanoma	Worst - Poor	0.685564454	0.553936233	0.369291	-0.33373
Melanoma	Worst - Good	0.685564454	0.465974714	0.388312	-0.28372
Melanoma	Poor - Good	0.685564454	0.468341984	0.468342	0.079438
Melanoma	Worst - Excellent	0.685564454	0.381103497	0.190552	-0.87586
Melanoma	Poor - Excellent	0.685564454	0.542222633	0.180741	-0.91255
Melanoma	Good - Excellent	0.685564454	0.98879212	0.164799	-0.97493
Merkel cell carcinoma	Worst - Poor	0.477200731	0.372647855	0.248432	0.679433
Merkel cell carcinoma	Worst - Good	0.477200731	0.439643486	0.439643	0.151873
Merkel cell carcinoma	Poor - Good	0.477200731	0.583443315	0.194481	-0.8615
Merkel cell carcinoma	Worst - Excellent	0.477200731	0.475514229	0.396262	-0.26303
Merkel cell carcinoma	Poor - Excellent	0.477200731	0.353209311	0.058868	-1.56435
Merkel cell carcinoma	Good - Excellent	0.477200731	0.492060067	0.24603	-0.68704
Multiple sclerosis	Worst - Poor	0.658399168	0.485440552	0.485441	0.036503
Multiple sclerosis	Worst - Good	0.658399168	0.506640006	0.33776	-0.41858
Multiple sclerosis	Poor - Good	0.658399168	0.674128618	0.22471	-0.75638
Multiple sclerosis	Worst - Excellent	0.658399168	0.500871246	0.250436	-0.67312
Multiple sclerosis	Poor - Excellent	0.658399168	0.696878869	0.116146	-1.19447
Multiple sclerosis	Good - Excellent	0.658399168	0.406551086	0.338793	-0.41576
Neoantigen	Worst - Poor	0.364616082	0.37593825	0.375938	-0.31617
Neoantigen	Worst - Good	0.364616082	0.354347173	0.236231	-0.71848
Neoantigen	Poor - Good	0.364616082	0.300736299	0.250614	-0.67256
Neoantigen	Worst - Excellent	0.364616082	0.313716296	0.104572	-1.25592
Neoantigen	Poor - Excellent	0.364616082	0.341054683	0.056842	-1.58185

Neoantigen	Good - Excellent	0.364616082	0.379013541	0.189507	-0.87972
Parkinson disease	Worst - Poor	0.54669682	0.469012509	0.390844	-0.27712
Parkinson disease	Worst - Good	0.54669682	0.28862926	0.19242	-0.86901
Parkinson disease	Poor - Good	0.54669682	0.485175633	0.161725	-0.98739
Parkinson disease	Worst - Excellent	0.54669682	0.335001842	0.167501	-0.96409
Parkinson disease	Poor - Excellent	0.54669682	0.742628361	0.123771	-1.15634
Parkinson disease	Good - Excellent	0.54669682	0.441435589	0.441436	-0.14733
Pollen allergen	Worst - Poor	0.157085399	0.118347174	0.019725	2.059472
Pollen allergen	Worst - Good	0.157085399	0.192536828	0.064179	1.520609
Pollen allergen	Poor - Good	0.157085399	0.153146252	0.127622	-1.1377
Pollen allergen	Worst - Excellent	0.157085399	0.118894857	0.079263	1.410045
Pollen allergen	Poor - Excellent	0.157085399	0.131652102	0.065826	-1.50762
Pollen allergen	Good - Excellent	0.157085399	0.381507518	0.381508	-0.30152
Psoriatic arthritis	Worst - Poor	0.649306935	0.398407078	0.332006	-0.43438
Psoriatic arthritis	Worst - Good	0.649306935	0.380025735	0.190013	-0.87785
Psoriatic arthritis	Poor - Good	0.649306935	0.343419037	0.228946	-0.74232
Psoriatic arthritis	Worst - Excellent	0.649306935	0.966892945	0.161149	-0.98975
Psoriatic arthritis	Poor - Excellent	0.649306935	0.524820993	0.17494	-0.93482
Psoriatic arthritis	Good - Excellent	0.649306935	0.430530541	0.430531	-0.17502
Rheumatoid arthritis	Worst - Poor	0.595231843	0.573435061	0.477863	-0.05552
Rheumatoid arthritis	Worst - Good	0.595231843	0.35785693	0.238571	-0.71091
Rheumatoid arthritis	Poor - Good	0.595231843	0.435444973	0.145148	-1.05747
Rheumatoid arthritis	Worst - Excellent	0.595231843	0.459077821	0.229539	-0.74037
Rheumatoid arthritis	Poor - Excellent	0.595231843	0.774669821	0.129112	-1.1306
Rheumatoid arthritis	Good - Excellent	0.595231843	0.487690971	0.487691	-0.03086
Ulcerative colitis	Worst - Poor	0.307849001	0.405722279	0.405722	-0.23856
Ulcerative colitis	Worst - Good	0.307849001	0.526282157	0.350855	-0.38301
Ulcerative colitis	Poor - Good	0.307849001	0.467811721	0.389843	-0.27973
Ulcerative colitis	Worst - Excellent	0.307849001	0.261694255	0.130847	-1.1224
Ulcerative colitis	Poor - Excellent	0.307849001	0.273800214	0.045633	-1.68875
Ulcerative colitis	Good - Excellent	0.307849001	0.235744896	0.078582	-1.41468
Yellow fever virus	Worst - Poor	0.739100638	0.500294908	0.416912	0.209799
Yellow fever virus	Worst - Good	0.739100638	0.438691329	0.438691	-0.15429

Yellow fever virus	Poor - Good	0.739100638	0.819646382	0.273215	-0.60312
Yellow fever virus	Worst - Excellent	0.739100638	0.489428525	0.326286	-0.45019
Yellow fever virus	Poor - Excellent	0.739100638	0.799793828	0.133299	-1.11093
Yellow fever virus	Good - Excellent	0.739100638	0.626224709	0.313112	-0.48705
