# Supplementary material for: Immune environment and antigen specificity of the T cell receptor repertoire of malignant ascites in ovarian cancer
Source: PLoS One. 2023 Jan 6;18(1):e0279590. doi: 10.1371/journal.pone.0279590 (PMC9821423; doi:10.1371/journal.pone.0279590)
Supplement: S6 Table — Red text indicated known CDR3β peptide (CASSLVNTEAFF) annotated by epithelial ovarian cancer and the TP53 neoantigen in McPAS. (PDF) [file pone.0279590.s013.pdf]

Supplementary Table S6. Productive T cell receptor rearrangements combined by the GLIPH algorithm in the specificity group with a known CDR3β peptide binding a *TP53* neoantigen. Red text indicated known CDR3β peptide (CASSLVNTEAFF) annotated by epithelial ovarian cancer and the *TP53* neoantigen in McPAS.

Index	Pattern	Fisher score	No. of subjects	No. of unique CDR3s	Final score	HLA score	VB score	Expansion score	Length score	Cluster size score	Type	ulTcRb	TcRb	V	J	TcRa	Sample
433	SL%NTE	9.90E-04	22	17	1.90E-08	1.00E+00	6.20E-01	3.30E-01	1.00E-03	9.50E-05	global-SL%NTE	CASSLtNTEAFF	CASSLTNTEAFF	TRBV07-09	TRBJ01-01	NA	12
433	SL%NTE	9.90E-04	22	17	1.90E-08	1.00E+00	6.20E-01	3.30E-01	1.00E-03	9.50E-05	global-SL%NTE	CASSLvNTEAFF	CASSLVNTEAFF	TRBV07-09	TRBJ01-01	NA	13
433	SL%NTE	9.90E-04	22	17	1.90E-08	1.00E+00	6.20E-01	3.30E-01	1.00E-03	9.50E-05	global-SL%NTE	CASSLtNTEAFF	CASSLTNTEAFF	TRBV05-01	TRBJ01-01	NA	14
433	SL%NTE	9.90E-04	22	17	1.90E-08	1.00E+00	6.20E-01	3.30E-01	1.00E-03	9.50E-05	global-SL%NTE	CASSLvNTEAFF	CASSLVNTEAFF	TRBV11-02	TRBJ01-01	NA	14
433	SL%NTE	9.90E-04	22	17	1.90E-08	1.00E+00	6.20E-01	3.30E-01	1.00E-03	9.50E-05	global-SL%NTE	CTSSLkNTEAFF	CTSSLKNTEAFF	TRBV01-01	TRBJ01-01	NA	14
433	SL%NTE	9.90E-04	22	17	1.90E-08	1.00E+00	6.20E-01	3.30E-01	1.00E-03	9.50E-05	global-SL%NTE	CASSLqNTEAFF	CASSLQNTEAFF	TRBV07-09	TRBJ01-01	NA	15
433	SL%NTE	9.90E-04	22	17	1.90E-08	1.00E+00	6.20E-01	3.30E-01	1.00E-03	9.50E-05	global-SL%NTE	CASSLgNTEAFF	CASSLGNTEAFF	TRBV12	TRBJ01-01	NA	15
433	SL%NTE	9.90E-04	22	17	1.90E-08	1.00E+00	6.20E-01	3.30E-01	1.00E-03	9.50E-05	global-SL%NTE	CSASlqNTEAFF	CSASLQNTEAFF	TRBV20	TRBJ01-01	NA	17
433	SL%NTE	9.90E-04	22	17	1.90E-08	1.00E+00	6.20E-01	3.30E-01	1.00E-03	9.50E-05	global-SL%NTE	CASSLqNTEAFF	CASSLQNTEAFF	TRBV28-01	TRBJ01-01	NA	17
433	SL%NTE	9.90E-04	22	17	1.90E-08	1.00E+00	6.20E-01	3.30E-01	1.00E-03	9.50E-05	global-SL%NTE	CASSLdNTEAFF	CASSLDNTEAFF	TRBV03	TRBJ01-01	NA	19
433	SL%NTE	9.90E-04	22	17	1.90E-08	1.00E+00	6.20E-01	3.30E-01	1.00E-03	9.50E-05	global-SL%NTE	CAWSlrNTEAFF	CAWSLRNTEAFF	TRBV30-01	TRBJ01-01	NA	19
433	SL%NTE	9.90E-04	22	17	1.90E-08	1.00E+00	6.20E-01	3.30E-01	1.00E-03	9.50E-05	global-SL%NTE	CASSLqNTEAFF	CASSLQNTEAFF	TRBV12	TRBJ01-01	NA	19
433	SL%NTE	9.90E-04	22	17	1.90E-08	1.00E+00	6.20E-01	3.30E-01	1.00E-03	9.50E-05	global-SL%NTE	CASSLqNTEAFF	CASSLQNTEAFF	TRBV07-02	TRBJ01-01	NA	20
433	SL%NTE	9.90E-04	22	17	1.90E-08	1.00E+00	6.20E-01	3.30E-01	1.00E-03	9.50E-05	global-SL%NTE	CASSLqNTEAFF	CASSLQNTEAFF	TRBV03	TRBJ01-01	NA	20
433	SL%NTE	9.90E-04	22	17	1.90E-08	1.00E+00	6.20E-01	3.30E-01	1.00E-03	9.50E-05	global-SL%NTE	CASSLgNTEAFF	CASSLGNTEAFF	TRBV07-03	TRBJ01-01	NA	20
433	SL%NTE	9.90E-04	22	17	1.90E-08	1.00E+00	6.20E-01	3.30E-01	1.00E-03	9.50E-05	global-SL%NTE	CASSLgNTEAFF	CASSLGNTEAFF	TRBV12	TRBJ01-01	NA	21
433	SL%NTE	9.90E-04	22	17	1.90E-08	1.00E+00	6.20E-01	3.30E-01	1.00E-03	9.50E-05	global-SL%NTE	CASSLvNTEAFF	CASSLVNTEAFF	TRBV05-04	TRBJ01-01	NA	22
433	SL%NTE	9.90E-04	22	17	1.90E-08	1.00E+00	6.20E-01	3.30E-01	1.00E-03	9.50E-05	global-SL%NTE	CASSLgNTEAFF	CASSLGNTEAFF	TRBV07-09	TRBJ01-01	NA	22
433	SL%NTE	9.90E-04	22	17	1.90E-08	1.00E+00	6.20E-01	3.30E-01	1.00E-03	9.50E-05	global-SL%NTE	CASSLpNTEAFF	CASSLPNTEAFF	TRBV12	TRBJ01-01	NA	22
433	SL%NTE	9.90E-04	22	17	1.90E-08	1.00E+00	6.20E-01	3.30E-01	1.00E-03	9.50E-05	global-SL%NTE	CASSLiNTEAFF	CASSLINTEAFF	TRBV28-01	TRBJ01-01	NA	22
433	SL%NTE	9.90E-04	22	17	1.90E-08	1.00E+00	6.20E-01	3.30E-01	1.00E-03	9.50E-05	global-SL%NTE	CASSLrNTEAFF	CASSLRNTEAFF	TRBV28-01	TRBJ01-01	NA	23
433	SL%NTE	9.90E-04	22	17	1.90E-08	1.00E+00	6.20E-01	3.30E-01	1.00E-03	9.50E-05	global-SL%NTE	CASSLgNTEAFF	CASSLGNTEAFF	TRBV05-01	TRBJ01-01	NA	23
433	SL%NTE	9.90E-04	22	17	1.90E-08	1.00E+00	6.20E-01	3.30E-01	1.00E-03	9.50E-05	global-SL%NTE	CASSLpNTEAFF	CASSLPNTEAFF	TRBV28-01	TRBJ01-01	NA	23
433	SL%NTE	9.90E-04	22	17	1.90E-08	1.00E+00	6.20E-01	3.30E-01	1.00E-03	9.50E-05	global-SL%NTE	CASSLeNTEAFF	CASSLENTEAFF	TRBV06-06	TRBJ01-01	NA	24
433	SL%NTE	9.90E-04	22	17	1.90E-08	1.00E+00	6.20E-01	3.30E-01	1.00E-03	9.50E-05	global-SL%NTE	CASSLqNTEAFF	CASSLQNTEAFF	TRBV11-02	TRBJ01-01	NA	25
433	SL%NTE	9.90E-04	22	17	1.90E-08	1.00E+00	6.20E-01	3.30E-01	1.00E-03	9.50E-05	global-SL%NTE	CASSLqNTEAFF	CASSLQNTEAFF	TRBV11-02	TRBJ01-01	NA	25
433	SL%NTE	9.90E-04	22	17	1.90E-08	1.00E+00	6.20E-01	3.30E-01	1.00E-03	9.50E-05	global-SL%NTE	CASSLtNTEAFF	CASSLTNTEAFF	TRBV02-01	TRBJ01-01	NA	27
433	SL%NTE	9.90E-04	22	17	1.90E-08	1.00E+00	6.20E-01	3.30E-01	1.00E-03	9.50E-05	global-SL%NTE	CAWSlmNTEAFF	CAWSLMNTEAFF	TRBV30-01	TRBJ01-01	NA	27

433	SL%NTE	9.90E-04	22	17	1.90E-08	1.00E+00	6.20E-01	3.30E-01	1.00E-03	9.50E-05	global-SL%NTE	CASSLmNTEAFF	CASSLMNTEAFF	TRBV14-01	TRBJ01-01	NA	30
433	SL%NTE	9.90E-04	22	17	1.90E-08	1.00E+00	6.20E-01	3.30E-01	1.00E-03	9.50E-05	global-SL%NTE	CASSLgNTEAFF	CASSLGNTEAFF	TRBV05-04	TRBJ01-01	NA	30
433	SL%NTE	9.90E-04	22	17	1.90E-08	1.00E+00	6.20E-01	3.30E-01	1.00E-03	9.50E-05	global-SL%NTE	CASSLwNTEAFF	CASSLWNTEAFF	TRBV19-01	TRBJ01-01	NA	31
433	SL%NTE	9.90E-04	22	17	1.90E-08	1.00E+00	6.20E-01	3.30E-01	1.00E-03	9.50E-05	global-SL%NTE	CASSLgNTEAFF	CASSLGNTEAFF	TRBV05-06	TRBJ01-01	NA	31
433	SL%NTE	9.90E-04	22	17	1.90E-08	1.00E+00	6.20E-01	3.30E-01	1.00E-03	9.50E-05	global-SL%NTE	CASSLmNTEAFF	CASSLMNTEAFF	TRBV12	TRBJ01-01	NA	35
433	SL%NTE	9.90E-04	22	17	1.90E-08	1.00E+00	6.20E-01	3.30E-01	1.00E-03	9.50E-05	global-SL%NTE	CASSLfNTEAFF	CASSLFNTEAFF	TRBV06	TRBJ01-01	NA	38
433	SL%NTE	9.90E-04	22	17	1.90E-08	1.00E+00	6.20E-01	3.30E-01	1.00E-03	9.50E-05	global-SL%NTE	CASSLvNTEAFF	CASSLVNTEAFF	TRBV07-08	TRBJ01-01	NA	40
433	SL%NTE	9.90E-04	22	17	1.90E-08	1.00E+00	6.20E-01	3.30E-01	1.00E-03	9.50E-05	global-SL%NTE	CAWSlmNTEAFF	CAWSLMNTEAFF	TRBV30-01	TRBJ01-01	NA	40
433	SL%NTE	9.90E-04	22	17	1.90E-08	1.00E+00	6.20E-01	3.30E-01	1.00E-03	9.50E-05	global-SL%NTE	CASSLiNTEAFF	CASSLINTEAFF	TRBV07-09	TRBJ01-01	NA	47
433	SL%NTE	9.90E-04	22	17	1.90E-08	1.00E+00	6.20E-01	3.30E-01	1.00E-03	9.50E-05	global-SL%NTE	CSASlqNTEAFF	CSASLQNTEAFF	TRBV20	TRBJ01-01	NA	48
433	SL%NTE	9.90E-04	22	17	1.90E-08	1.00E+00	6.20E-01	3.30E-01	1.00E-03	9.50E-05	global-SL%NTE	CASSLeNTEAFF	CASSLENTEAFF	TRBV02-01	TRBJ01-01	NA	48
433	SL%NTE	9.90E-04	22	17	1.90E-08	1.00E+00	6.20E-01	3.30E-01	1.00E-03	9.50E-05	global-SL%NTE	CASSLgNTEAFF	CASSLGNTEAFF	TRBV05-01	TRBJ01-01	NA	48
433	SL%NTE	9.90E-04	22	17	1.90E-08	1.00E+00	6.20E-01	3.30E-01	1.00E-03	9.50E-05	global-SL%NTE	CAWSlkNTEAFF	CAWSLKNTEAFF	TRBV30-01	TRBJ01-01	NA	49
433	SL%NTE	9.90E-04	22	17	1.90E-08	1.00E+00	6.20E-01	3.30E-01	1.00E-03	9.50E-05	global-SL%NTE	CASSLgNTEAFF	CASSLGNTEAFF	TRBV12	TRBJ01-01	NA	49
433	SL%NTE	9.90E-04	22	17	1.90E-08	1.00E+00	6.20E-01	3.30E-01	1.00E-03	9.50E-05	global-SL%NTE	CASSLeNTEAFF	CASSLENTEAFF	TRBV11-03	TRBJ01-01	NA	50
