# Supplementary material for: Immune environment and antigen specificity of the T cell receptor repertoire of malignant ascites in ovarian cancer
Source: PLoS One. 2023 Jan 6;18(1):e0279590. doi: 10.1371/journal.pone.0279590 (PMC9821423; doi:10.1371/journal.pone.0279590)
Supplement: S7 Table — Neoantigens found by A, McPAS and B, VDJdb annotation among clustered CDR3β peptides associated with excellent or poor/worst prognosis. (PDF) [file pone.0279590.s014.pdf]

Supplementary Table S7. Neoantigens found by (**A**) McPAS and (**B**)VDJdb annotation among clustered CDR3β peptides associated with excellent or poor/worst prognosis.

A.

	**Associated with excellent prognosis**	**Associated with poor/worst prognosis**
**CD4+ T cells**	DHX33-LLA-M4I<br>HTR1F-9-V1M<br>OR2T1-FLN-F5L<br>OR5M3-KMV-T8N<br>MRM1-9<br>COL18A1-VLL-S8F<br>ST6GALNAC2-LLF-Y6H<br>ITIH6-RLG<br>PIGN-FLT-P7H<br>FNDC3B<br>LCP1-NLF<br>TBX3-GMG	PLXNB1-VLF<br>GFAP-NLA<br>CMV-MLN<br>PGM5<br>DHX33-LLA-K5T<br>MLL2<br>COL18A1<br>TRPC1-MLL-Q5H<br>MLL2-L8H<br>LCP1-NLF-PL<br>TTLL12-KLP
**CD8+ T cells**	Melan-A/MART-1<br>OR2T1-FLN-F5L<br>LTAg<br>OR5M3-KMV-T8N<br>CD1-LLG<br>OR14C36-FML-V6L<br>GANAB-ALY-S5F<br>DHX33-LLA-M4I<br>KAT6A-KLS<br>HTR1F-9-V1M<br>CMV-MLN<br>TEAD1-VLE-L8F<br>Melan-A A27L<br>ATP6AP1-KLG-G3W	HAUS3-ILN-T7A<br>NSDHL-ILT-A9V<br>MEGEA10-GLY<br>FNDC3B<br>Melan-A/MART-1

B.

	**Associated with excellent prognosis**	**Associated with poor/worst prognosis**
**CD4+ T cells**	PORCN<br>AKAP13	GCN1L1<br>TKT<br>ZDBF2
**CD8+ T cells**	PLA2G6<br>PMEL<br>NDC1	SLC30A8<br>IGF2BP2<br>GCN1L1<br>BST2<br>NY-ESO-1
